# Supplementary material for: Evaluating acute image ordering for real-world patient cases via language model alignment with radiological guidelines
Source: Commun Med (Lond). 2025 Aug 4;5:332. doi: 10.1038/s43856-025-01061-9 (PMC12322208; doi:10.1038/s43856-025-01061-9)
Supplement: Supplementary file 2 — Supplementary Information [file 43856_2025_1061_MOESM2_ESM.pdf]

Supplementary Information

Evaluating Acute Image Ordering for Real-World Patient Cases
via Language Model Alignment with Radiological Guidelines

Michael S. Yao[1,2], Allison Chae[2], Piya Saraiya[3], Charles E. Kahn, Jr.[2,3], Walter R. Witschey[2,3], James C. Gee[3], Hersh Sagreiya[2,3,†], Osbert Bastani[4,†,*]

Affiliations:
[1]Department of Bioengineering
[2]Perelman School of Medicine
[3]Department of Radiology
[4]Department of Computer and Information Science
University of Pennsylvania
Philadelphia, PA, USA 19104

[†]These authors jointly supervised this work.

*Correspondence should be addressed to O.B.:
Email: obastani@seas.upenn.edu
Phone: 215-898-8560
3330 Walnut St, Philadelphia, PA, 19104

Table of Contents

Supplementary Figures 1 – 13 ..... S1 – S13
Supplementary Tables 1 – 12 ..... S14 – S25
Supplementary Results A: Additional Prospective Clinician-AI Study Results S26
Supplementary Methods B: Large Language Model System and User Prompts ..... S27 – S32
Supplementary Results C: Sample Chain-of-Thought (COT) Reasoning Traces..... S33 – S35
Supplementary Results D: Example Curation of Long-Form Patient Scenarios ..... S36 – S45
Supplementary References S46

SUPPLEMENTARY FIGURES

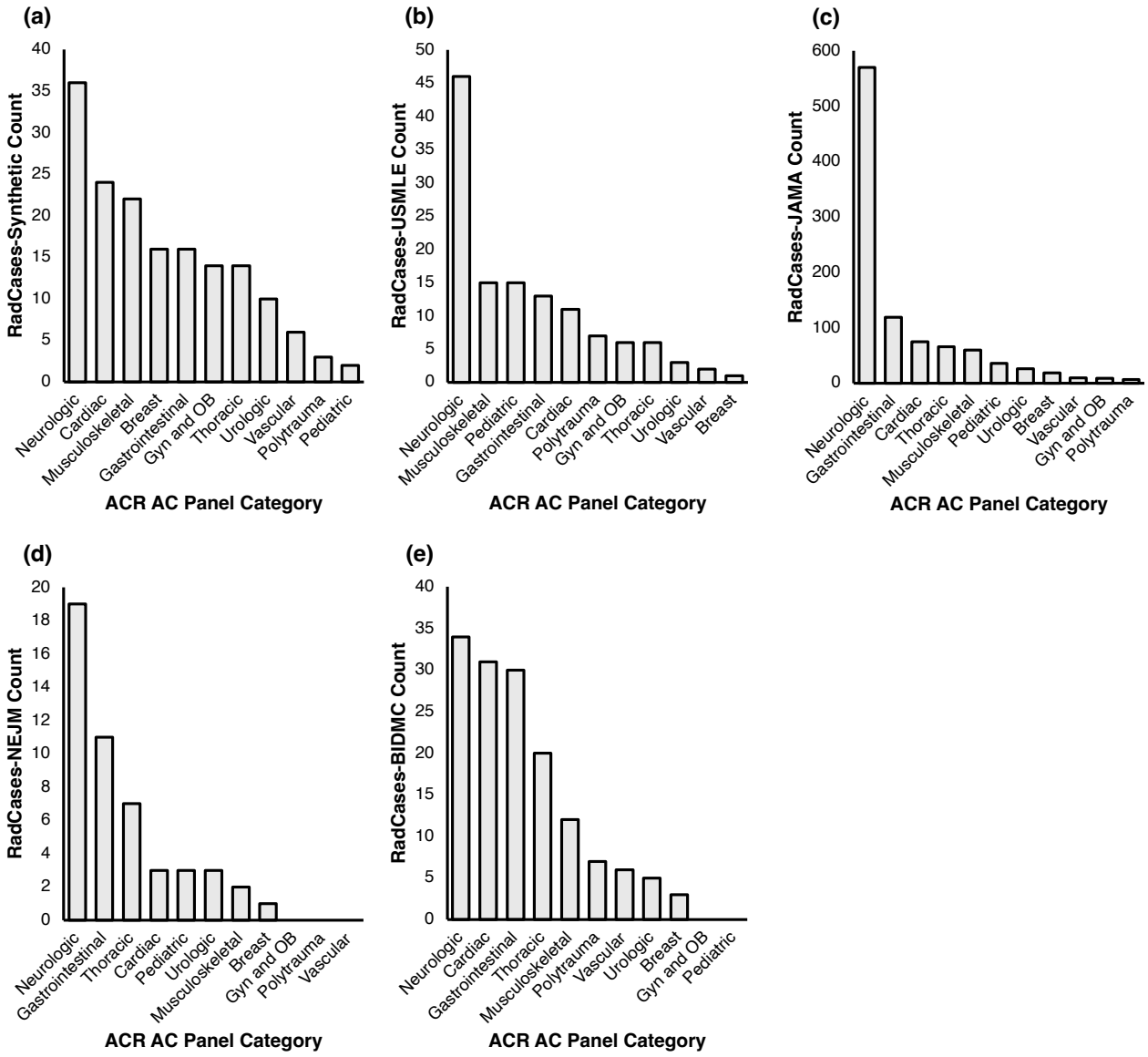

Supplementary Figure 1: ACR AC Panel Counts in the RadCases Dataset. As of June 2024, there are 224 ACR AC Topics that each has at least one assigned parent ACR AC Panel. Panels are more general categories for conditions, and there are 11 to date: Breast, Cardiac, Gastrointestinal, Gyn and OB, Musculoskeletal, Neurologic, Pediatric, Polytrauma, Thoracic, Urologic, Vascular. To illustrate the distribution of conditions present in the RadCases dataset, we plot the counts of each of these 11 parent ACR AC Panels for the **(a) Synthetic**; **(b) USMLE**; **(c) JAMA**; **(d) NEJM**; and **(e) BIDMC** subsets of the RadCases dataset.

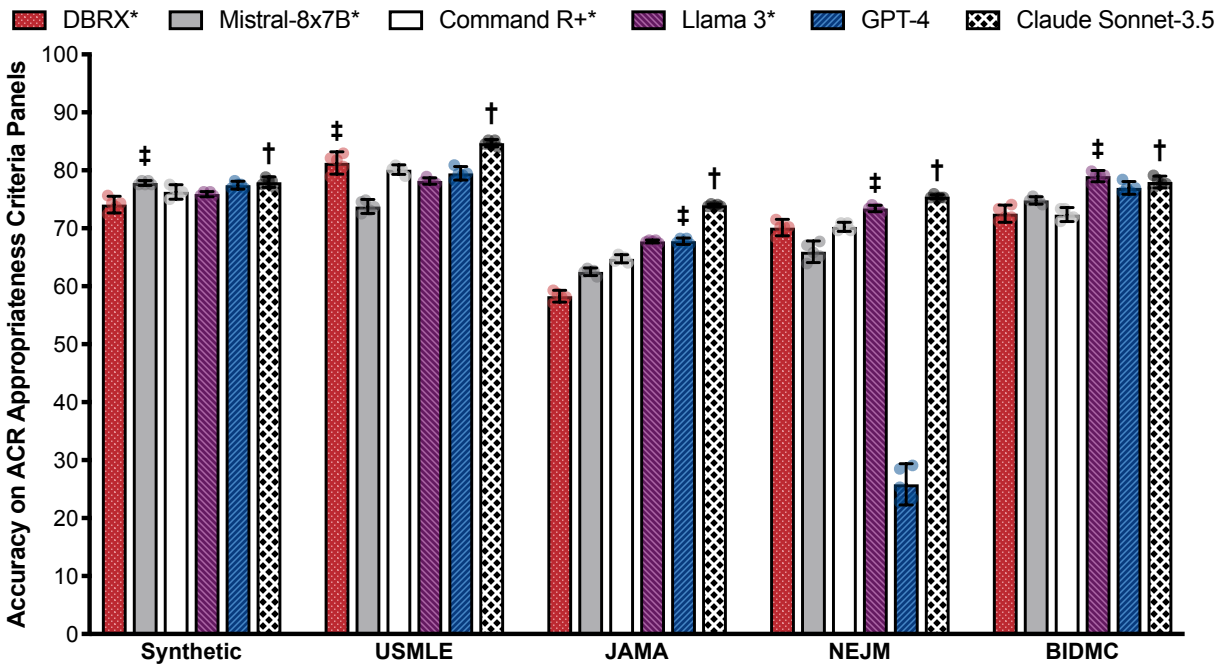

Supplementary Figure 2: Baseline LLM Performance on ACR AC Panel Classification Using the RadCases Dataset. In **Figure 2b,** we evaluate six state-of-the-art large language models (LLMs) on their ability to correctly assign 1 of 224 ACR AC Topics to an input one-liner. Here, we include analogous results on the related ACR AC Panel classification task, which queries an LLM to correctly assign 1 of 11 ACR AC Panels to an input one-liner. Because ACR AC Panels are more coarse-grained than ACR AC Topics, a language model's accuracy on this task can help assess the model's ability to identify the general body part or organ system affected by pathophysiology. However, accuracy on this task is not helpful for ordering imaging studies, as there is no clear method for assigning a "correct" imaging study given only an ACR AC Panel. Open-source models are identified by an asterisk, and the best (second best) performing model for a RadCases dataset partition is identified by a dagger (double dagger). Error bars represent ± 95% CI over n = 5 independent experimental runs.

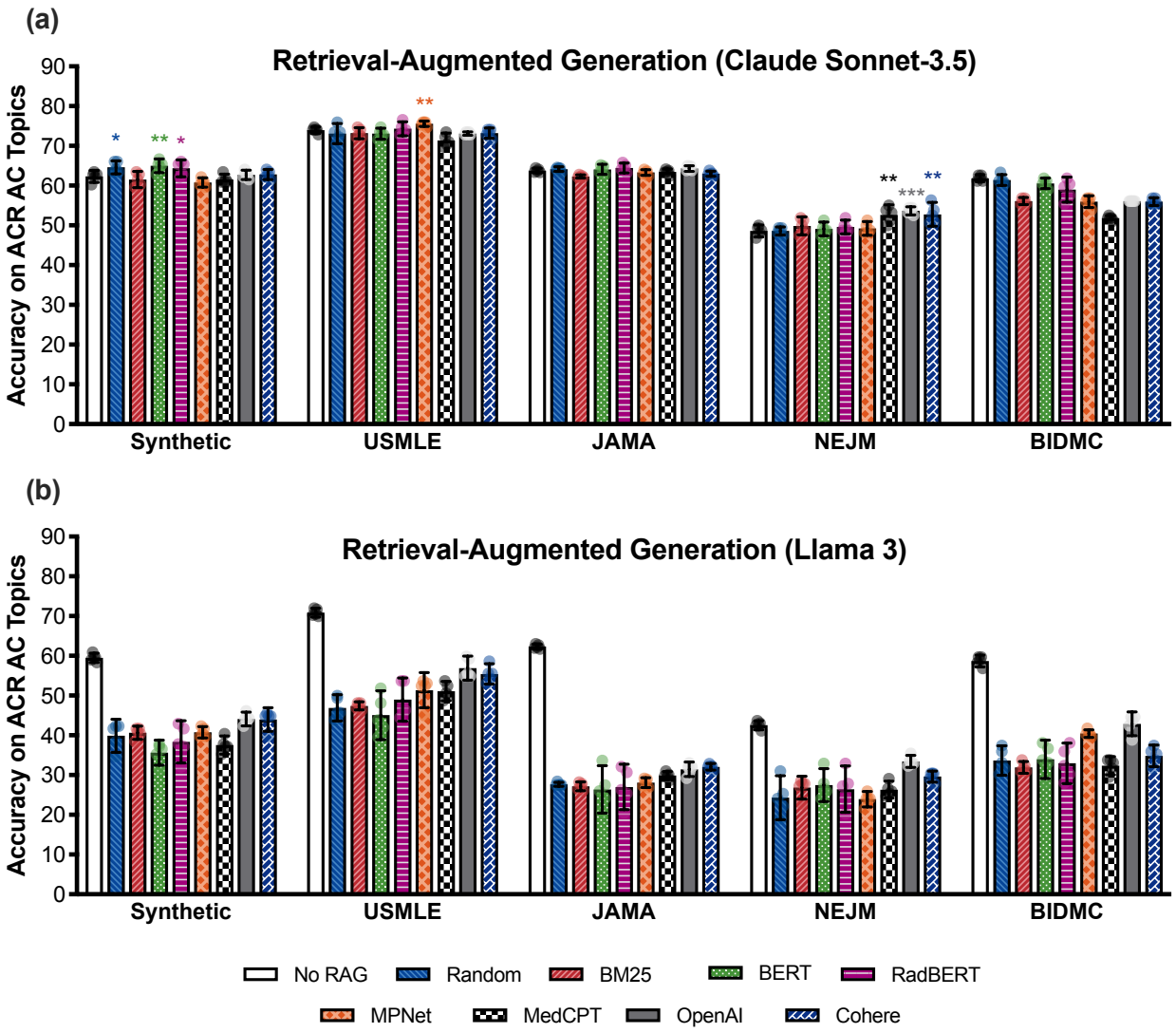

Supplementary Figure 3: Retrieval-Augmented Generation (RAG) Performance versus Retriever Algorithm. To optimize RAG for LLM accuracy on the ACR AC Topic classification task, we evaluated 8 different retrieval algorithms to use in RAG: (1) **Random**, which randomly retrieves documents from the corpus over a uniform probability distribution; (2) Okapi **BM25** bag-of-words retriever; (3) **BERT** and (4) **MPNet** trained on unlabeled, natural language text; (5) **RadBERT** from fine-tuning BERT on radiology text reports; (6) **MedCPT** leveraging a transformer trained on PubMed search logs; and (7) **OpenAI** (text-embedding-3-large) and (8) **Cohere** (cohere.embed-english-v3) embedding models from OpenAI and Cohere for AI, respectively. Using **(a)** Claude Sonnet-3.5 and **(b)** Llama 3, we retrieve 8 documents from the ACR AC narrative guidelines corpus using each retriever and compare each method against the baseline ACR AC Topic accuracy achieved by each model. Error bars represent ± 95% CI over *n* = 5 independent experimental runs.

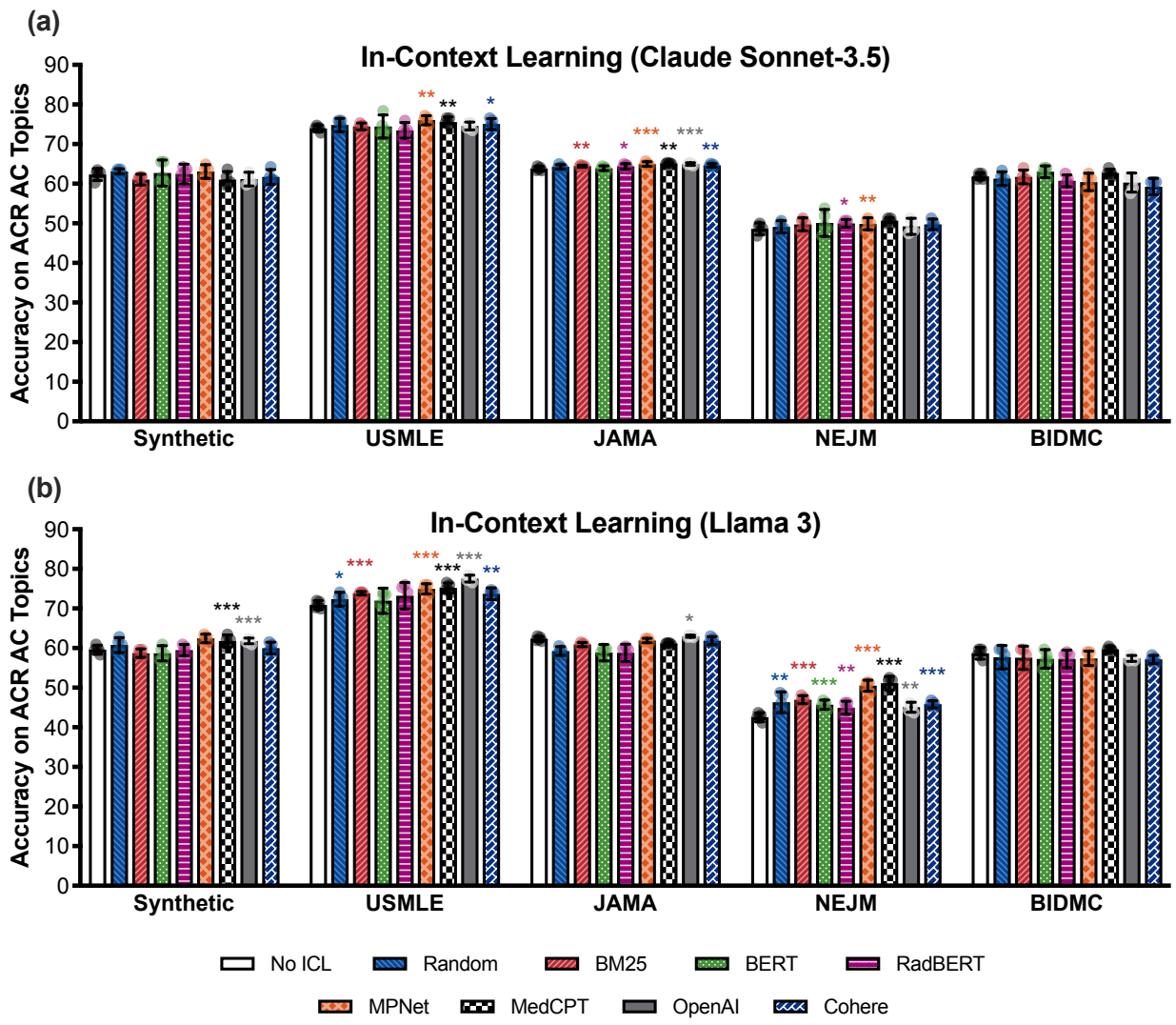

Supplementary Figure 4: In-Context Learning (ICL) Performance versus Retriever Algorithm. To optimize ICL for LLM accuracy on the ACR AC Topic classification task, we evaluated 8 different retrieval algorithms to use in ICL, identical to those explored in RAG (see caption of **Supplementary Fig. 3**). Using **(a)** Claude Sonnet-3.5 and **(b)** Llama 3, we retrieve 4 example one-liner/Topic pairs from the RadCases-Synthetic dataset corpus using each retriever and compare each method against baseline ACR AC Topic accuracy achieved by each model. To avoid data leakage, a separate synthetic dataset (generated from Meta Llama 2 instead of OpenAI GPT-3.5) was used as the retrieval corpus to evaluate ICL on the RadCases-Synthetic dataset. Error bars represent ± 95% CI over *n* = 5 independent experimental runs.

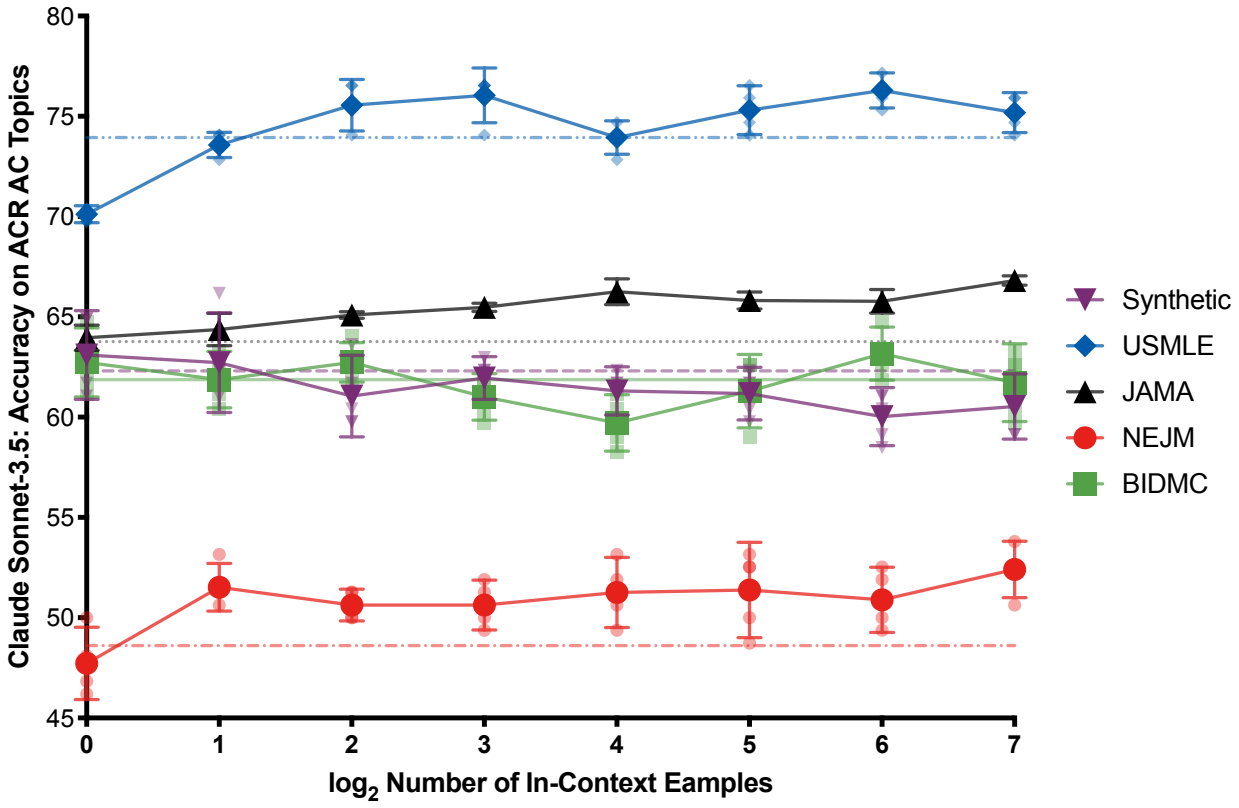

Supplementary Figure 5: In-Context Learning (ICL) Performance versus Retriever Budget. Using the subjectively best retriever algorithm evaluated in **Supplementary Fig. 4** (i.e. the MedCPT retriever), we evaluated the effect of changing the number of ICL examples retrieved by the retriever to be passed as context to Claude Sonnet-3.5. The purple dashed, blue double dotted-dashed, black dotted, red dotted-dashed, and green solid horizontal lines correspond to the baseline, no-ICL accuracy scores of Claude Sonnet-3.5 on the Synthetic, USMLE, JAMA, NEJM, and BIDMC subsets of the RadCases dataset, respectively. For the USMLE, JAMA, and NEJM subsets, we find that the performance of the model increases as the number of ICL examples increases from 1 to 128. To avoid data leakage, a separate synthetic dataset (generated from Meta Llama 2 instead of OpenAI GPT-3.5) was used as the retrieval corpus to evaluate ICL on the RadCases-Synthetic dataset. Error bars represent ± 95% CI over n = 5 independent experimental runs.

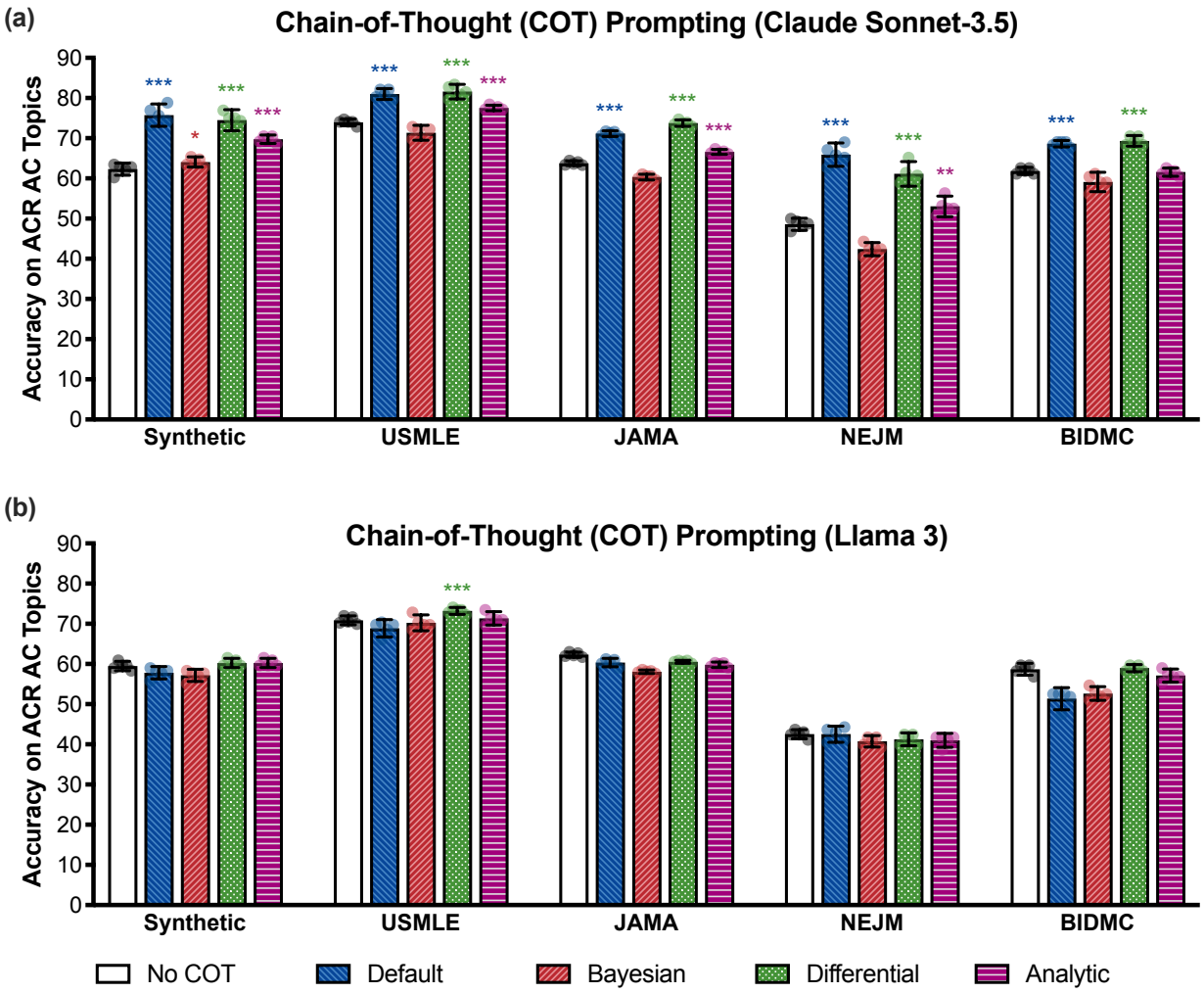

Supplementary Figure 6: Chain-of-Thought (COT) Prompting Performance versus Reasoning Algorithm. To optimize COT prompting to improve LLM accuracy on the ACR AC Topic classification task for both **(a)** Claude Sonnet-3.5 and **(b)** Llama 3, we investigated 4 different COT reasoning methods: (1) **Default** reasoning, which does not specify any particular reasoning strategy for the LLM to use; (2) **Differential** diagnosis reasoning, which encourages the model to reason through a differential diagnosis to arrive at a final prediction; (3) **Bayesian** reasoning, which encourages the model to approximate Bayesian posterior updates over the space of ACR AC Topics based on the clinical patient presentation; and (4) **Analytic** reasoning, which encourages the model to reason through the underlying pathophysiology. The exact prompts used in each of these reasoning methods are included in **Supplementary Methods B**. We compare each method against the baseline ACR AC Topic accuracy achieved by each model. Error bars represent ± 95% CI over *n* = 5 independent experimental runs.

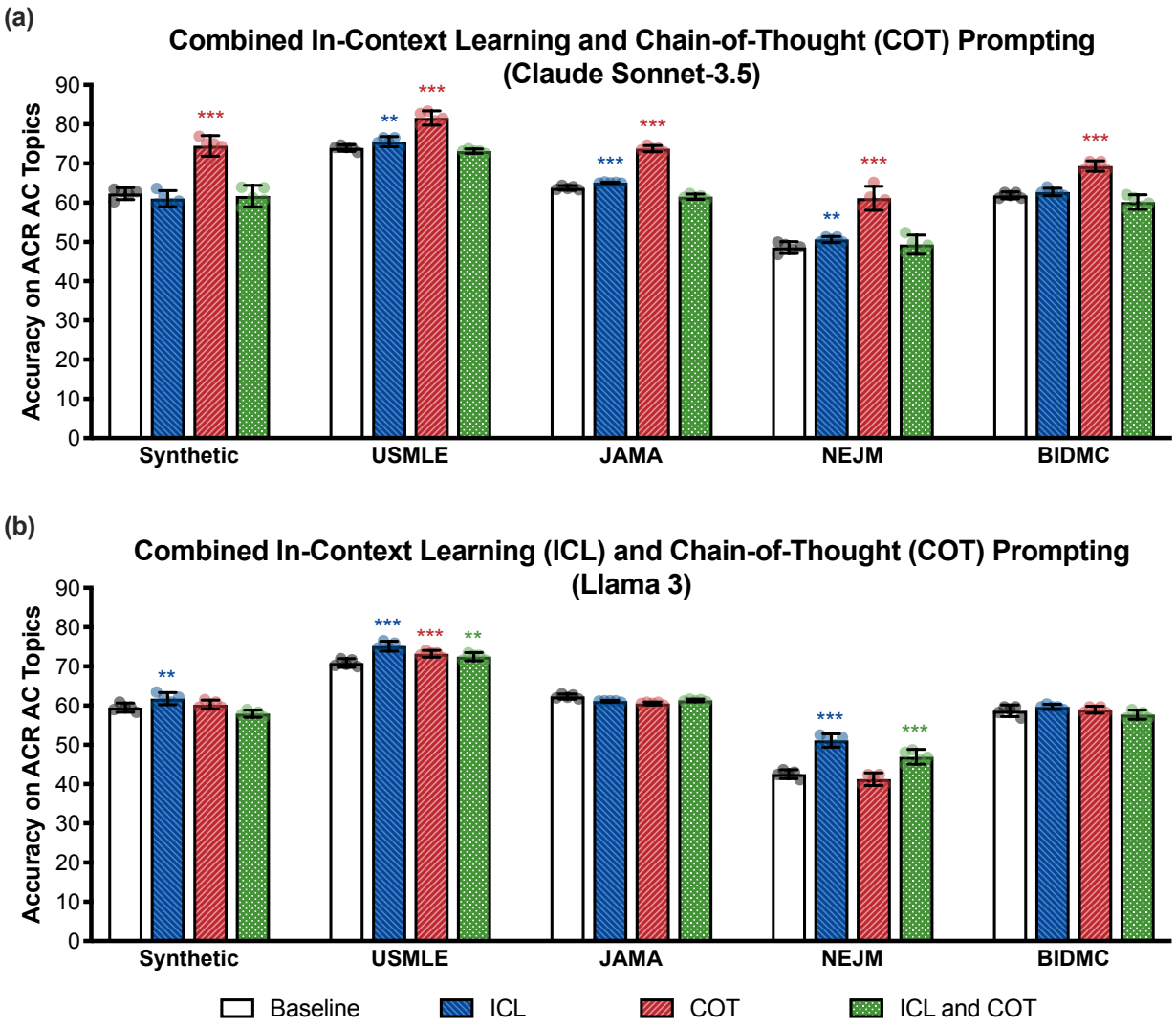

Supplementary Figure 7: Combining In-Context Learning (ICL) and Chain-of-Thought (COT) Prompting Strategies. In **Supplementary Figures 4 and 6**, we found that ICL (using the MedCPT retriever) and COT (using the Default reasoning strategy) were effective prompting strategies to improve the performance of Claude Sonnet-3.5 and/or Llama 3. We combine these two strategies together to investigate whether their combination could further improve model performance of both **(a)** Claude Sonnet-3.5 and **(b)** Llama 3. We compare each method against the baseline, ICL-only, and COT-only ACR AC Topic accuracy achieved by each model. Error bars represent ± 95% CI over *n* = 5 independent experimental runs.

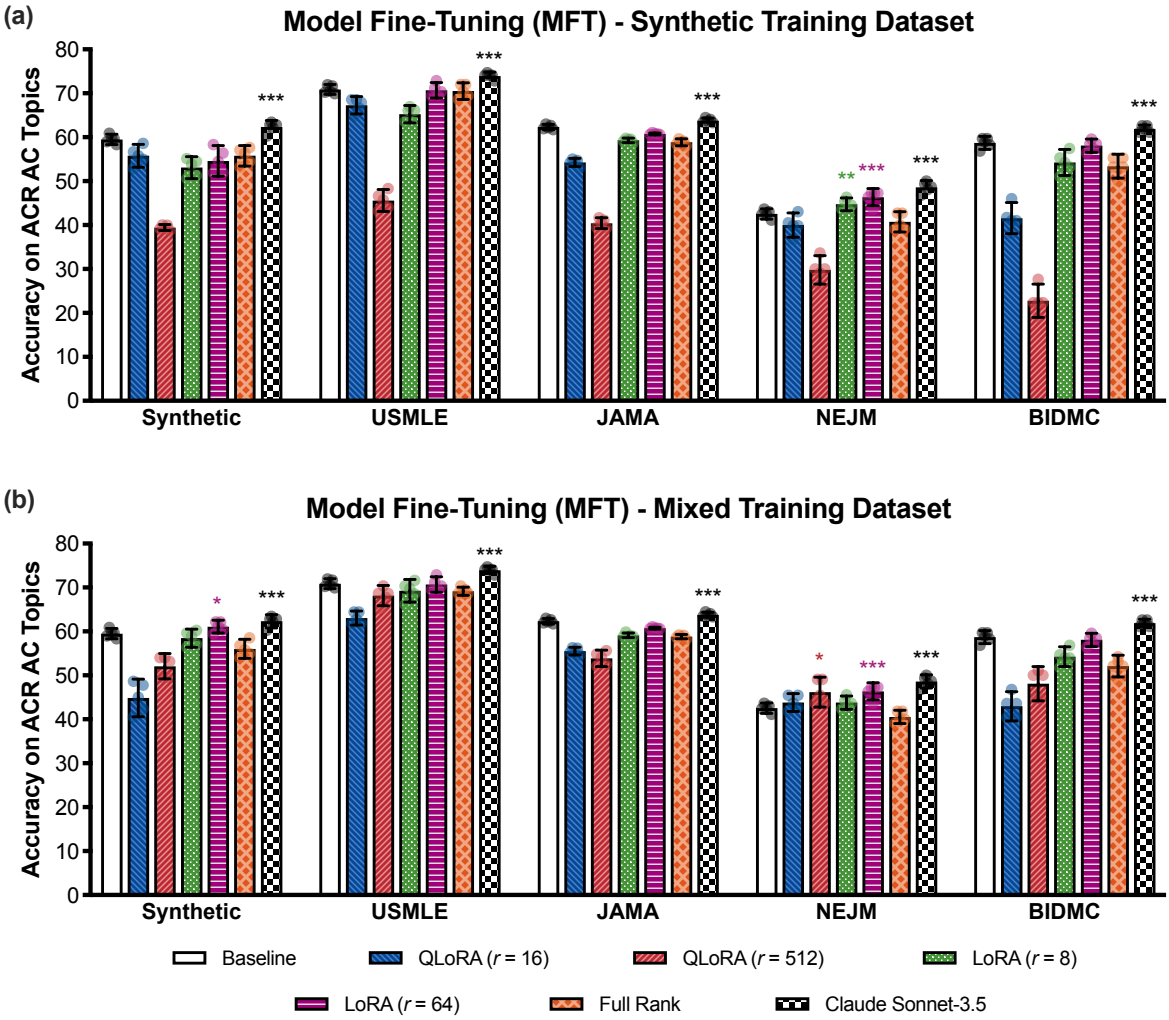

Supplementary Figure 8: Model Fine-Tuning (MFT) Algorithm Evaluation with Llama 3. We evaluate 5 different fine-tuning experimental setups in our MFT experiments: quantized low-rank adaptation (**QLoRA**) with a rank of (1) r = 16 and (2) r = 512; low-rank adaptation (**LoRA**) with a rank of (3) r = 8 and (4) r = 64; and (5) **Full Rank** model fine-tuning. We use an α scaling value of 8 for all QLoRA and LoRA experiments. To construct the MFT training dataset, we use either **(a)** all n = 156 labeled one-liners from the RadCases-**Synthetic** dataset; or **(b)** a **Mixed** dataset including 50 randomly selected cases from each of the 5 RadCases dataset subsets for a total of n = 250 labeled one-liners. The first scenario simulates a setting where we can only fine-tune models on synthetically generated data due to privacy concerns, and the latter scenario simulates a setting where we are able to train on real patient data sampled from the relevant distribution(s) of interest. To avoid data leakage, a separate synthetic dataset (generated from Meta Llama 2 instead of OpenAI GPT-3.5) was used to fine-tune the base model for evaluation on the RadCases-Synthetic dataset. Error bars represent ± 95% CI over n = 5 independent experimental runs.

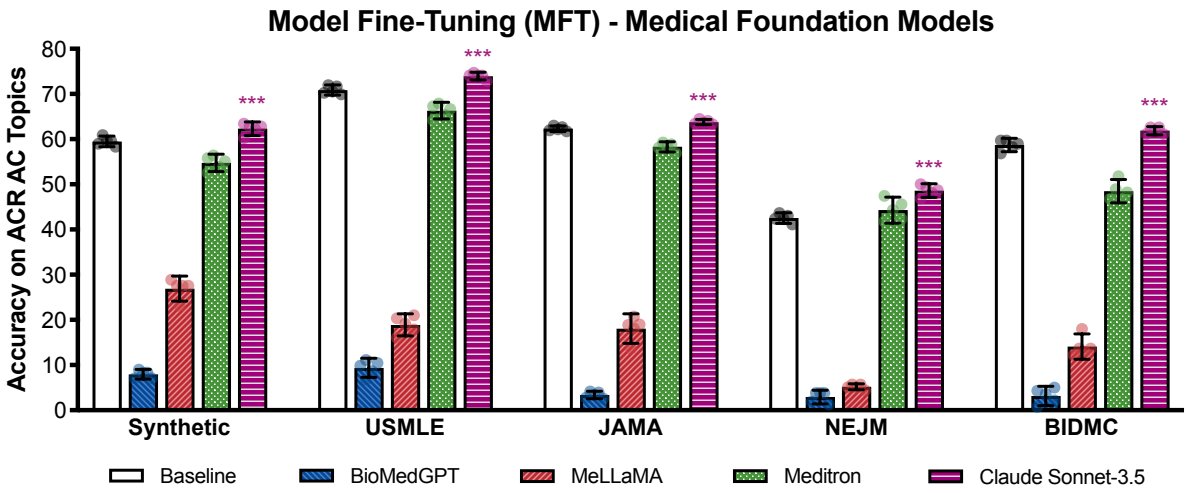

Supplementary Figure 9: Evaluating Medical Foundation Models Fine-Tuned on Llama LLMs. Separate from the results presented in **Supplementary Figure 8**, an alternative approach to model fine-tuning is instead to leverage language models fine-tuned on large corpora of domain-specific medical text. Examples of such foundation models include BioMedGPT-7B,[1] MeLLaMA-70B,[2] and Meditron-70B.[3] We evaluate their accuracies in predicting ACR AC Topic labels for the RadCases datasets; none of the three medical foundation models outperformed the base Meta Llama 3 70B model with statistical significance. Our results are consistent with findings reported by prior work[4-7] and highlight the challenge in fine-tuning language models specifically for RadCases and other medical tasks. Error bars represent ± 95% CI over n = 5 independent experimental runs.

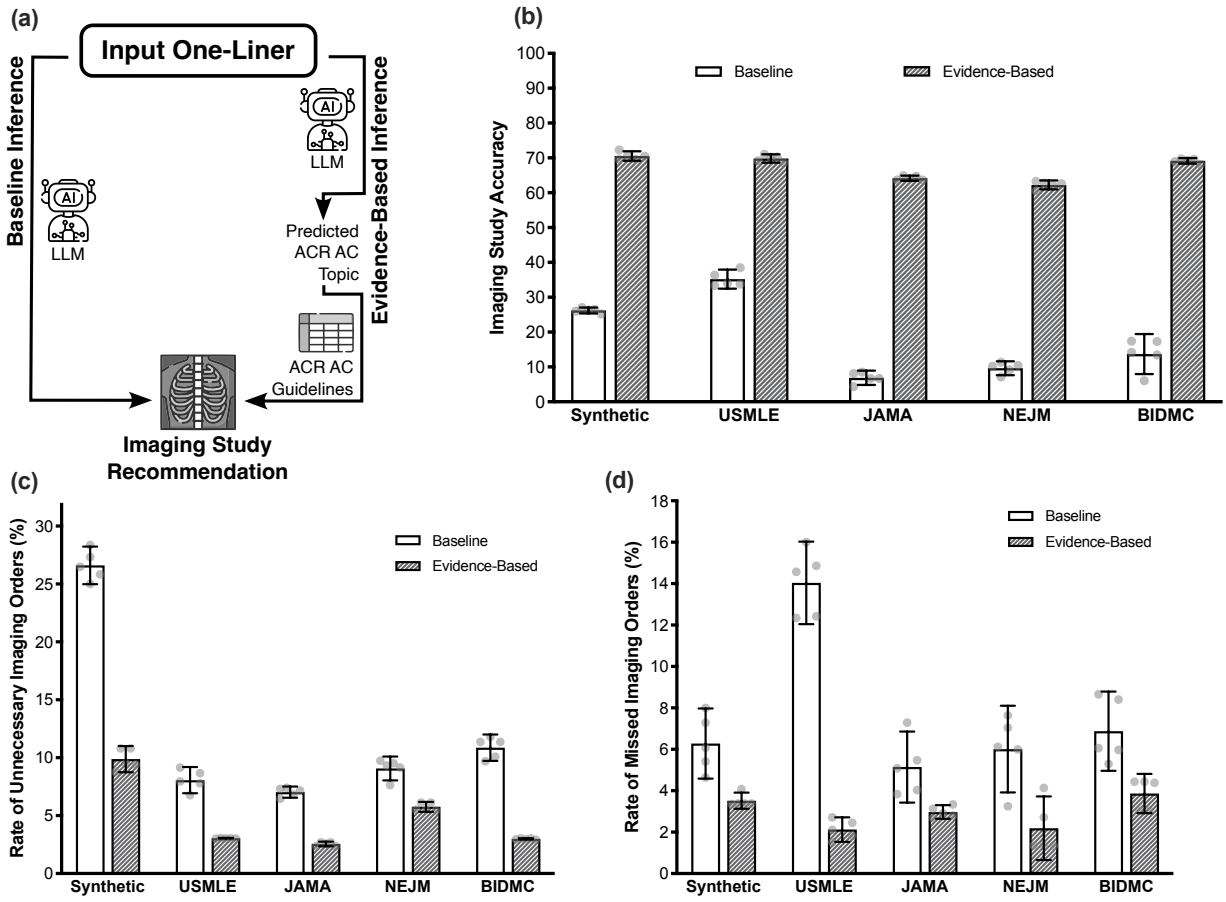

Supplementary Figure 10: Comparison of Baseline and Evidence-Based Inference Pipelines with Llama 3. (a) Using our evidence-based inference pipeline identical to that in **Fig. 3a**, we query Llama 3 to predict the ACR AC Topic most relevant to an input patient one-liner, and programmatically refer to the ACR AC guidelines to make the final recommendation for diagnostic imaging (**Evidence-Based**). An alternative approach is the baseline inference pipeline where we query the LLM to recommend a diagnostic imaging study directly without using the ACR AC (**Baseline**). Because there was no consistently optimal prompting or fine-tuning strategy that outperformed Baseline prompting in **Fig. 3c**, we only empirically evaluated the base Evidence-Based inference strategy (i.e., without any prompt optimizations) here. **(b)** Our evidence-based pipeline significantly outperforms the baseline pipeline by up to 57.3% (two-sample, one-tailed, homoscedastic *t*-test; Synthetic $p = 5.06 \times 10^{-13}$; USMLE $p = 4.64 \times 10^{-10}$; JAMA $p = 6.52 \times 10^{-13}$; NEJM $p = 2.70 \times 10^{-12}$; BIDMC $p = 2.18 \times 10^{-9}$). At the same time, our method also reduces the rates of both **(c)** unnecessary (two-sample, one-tailed, homoscedastic *t*-test; Synthetic $p = 5.75 \times 10^{-9}$; USMLE $p = 8.94 \times 10^{-7}$; JAMA $p = 5.18 \times 10^{-9}$; NEJM $p = 1.74 \times 10^{-5}$; BIDMC $p = 2.79 \times 10^{-8}$) and **(d)** missed imaging orders (two-sample, one-tailed, homoscedastic *t*-test; Synthetic $p = 1.13 \times 10^{-3}$; USMLE $p = 1.23 \times 10^{-7}$; JAMA $p = 4.35 \times 10^{-3}$; NEJM $p = 1.76 \times 10^{-3}$; BIDMC $p = 2.20 \times 10^{-3}$) compared to the baseline. Error bars represent ± 95% CI over $n = 5$ independent experimental runs.

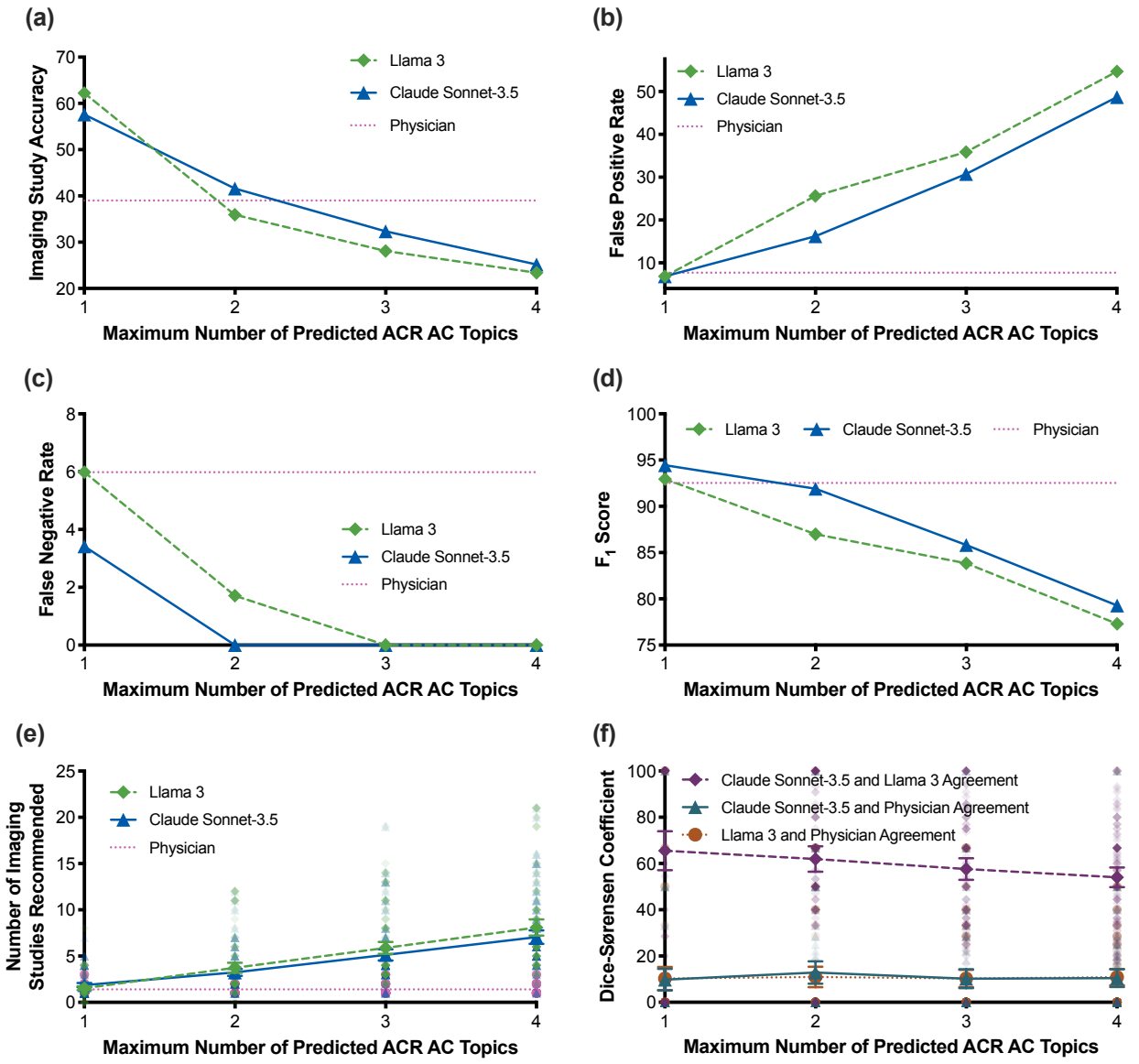

Supplementary Figure 11: Ablation Study – Number of ACR AC Topic Predictions in Retrospective Study of Physician-Ordered versus LLM-Ordered Imaging Studies. In **Figure 5**, we show the results of our retrospective study evaluating diagnostic imaging orders of LLMs and physicians—both Claude Sonnet-3.5 and Llama 3 were prompted to predict the single $m = 1$ best ACR AC Topic for an input patient description. Here, we vary the maximum number m of ACR AC Topic predictions requested from each language model along the *x*-axis. We compare the **(a)** accuracy scores; **(b)** false positive rates (i.e., the rate at which a patient received at least one unnecessary imaging recommendation); **(c)** false negative rates (i.e., the rate at which a patient should have received an imaging workup but did not receive one); **(d)** F1 scores; **(e)** number of recommended imaging studies; and **(f)** similarity between the imaging studies ordered by Claude Sonnet-3.5, Llama 3, and physicians as a function of m.

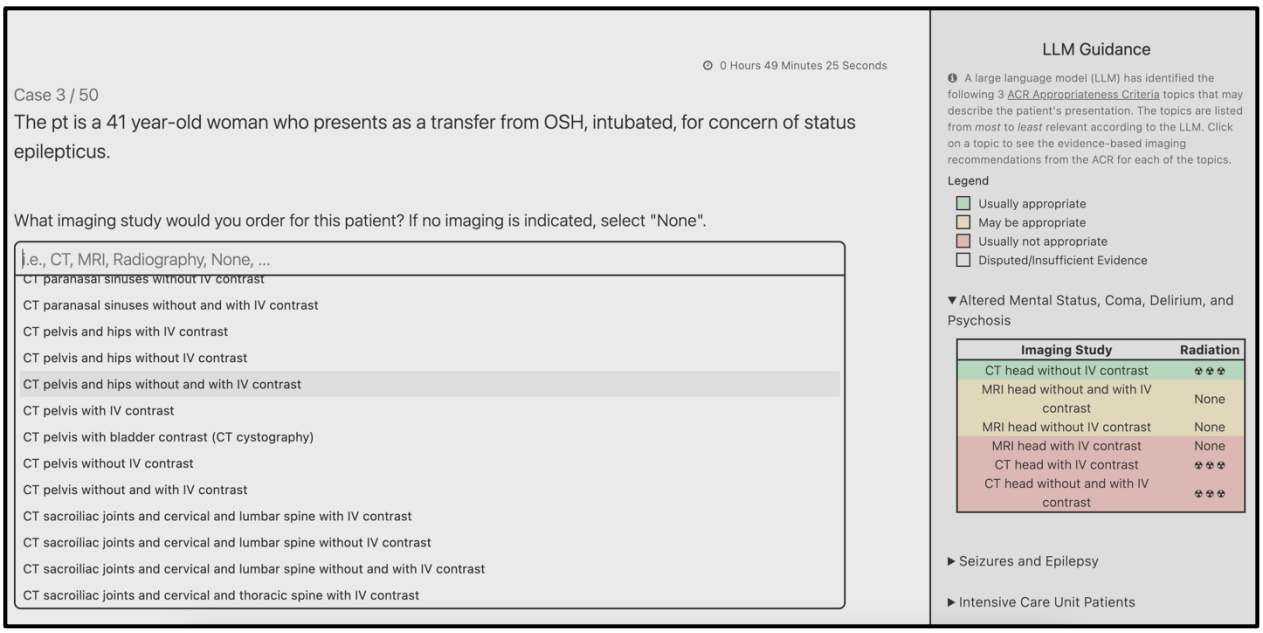

Supplementary Figure 12: User Interface for Prospective Study. The LLM is asked to predict up to three ACR Appropriateness Criteria (AC) Topics that may be relevant for the patient case, and the table of corresponding ACR AC recommendations is displayed for user reference. In questions where LLM guidance is not made available, the right column does not show any recommendations and instead displays the message “LLM guidance is not available for this patient scenario.”

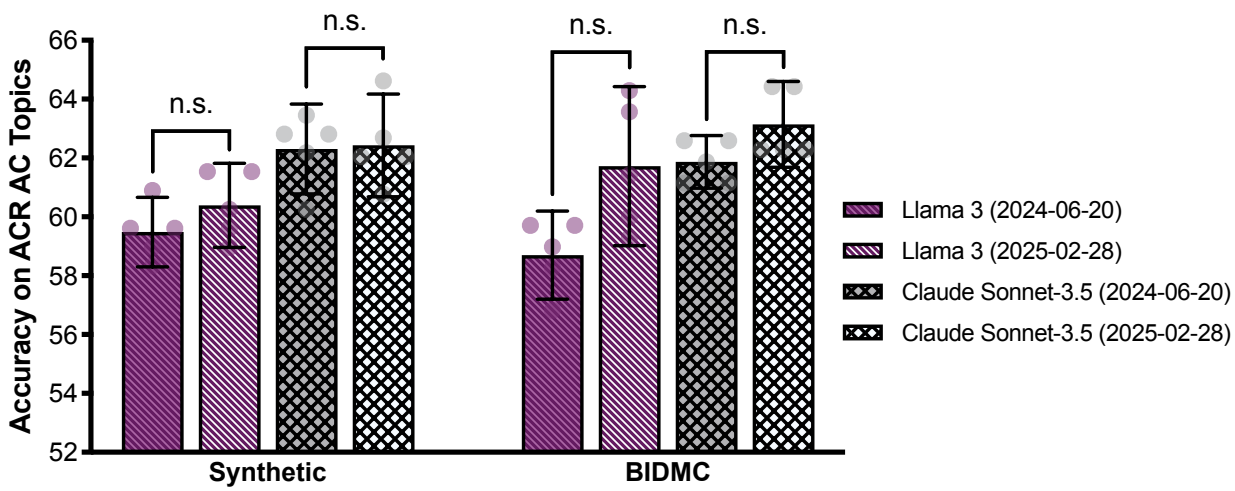

Supplementary Figure 13: Interrogating Model Performance Over Time. As of June 2024, when our study was initially conducted, there were 224 diagnostic imaging topics in the ACR Appropriateness Criteria (AC). However, as the guidelines are revised over time and new topics are added, the performance of language models using newer versions of the ACR AC may change. To investigate this, we re-labeled the Synthetic and BIDMC partitions of the RadCases dataset using the updated ACR AC in February 2025, with 9 new diagnostic imaging topics added to the ACR AC since June 2024. We repeated our initial experiment in **Figure 2** using **(a)** Llama 3 and **(b)** Claude Sonnet-3.5. Our results demonstrate that there is no statistically significant difference in model performance depending on whether the June 2024 ACR AC or February 2025 ACR AC was used. Error bars represent ± 95% CI over *n* = 5 independent experimental runs.

SUPPLEMENTARY TABLES

**Synthetic Subset ($n_{\text{total}} = 156$)**	**Count (%)**
Cardiac > Chest Pain-Possible Acute Coronary Syndrome	10 (6.41%)
Cardiac/Vascular > Suspected Pulmonary Embolism	8 (5.13%)
Gyn and OB > Acute Pelvic Pain in the Reproductive Age Group	6 (3.85%)
Neurologic > Low Back Pain	6 (3.85%)
Breast > Breast Pain	5 (3.21%)

**USMLE Subset ($n_{\text{total}} = 164$)**	**Count (%)**
Neurologic > Altered Mental Status, Coma, Delirium, and Psychosis	17 (10.4%)
Neurologic > Headache	12 (7.32%)
Cardiac > Chest Pain-Possible Acute Coronary Syndrome	9 (5.49%)
Polytrauma > Major Blunt Trauma	9 (5.49%)
Cardiac > Dyspnea-Suspected Cardiac Origin	8 (4.88%)

**JAMA Subset ($n_{\text{total}} = 971$)**	**Count (%)**
Neurologic > Orbits, Vision, and Visual Loss	280 (28.8%)
Neurologic > Neck Mass/Adenopathy	48 (4.94%)
Neurologic > Staging and Post-Therapy Assessment of Head and Neck Cancer	42 (4.33%)
Neurologic > Headache	31 (3.19%)
Gastrointestinal > Acute Nonlocalized Abdominal Pain	28 (2.88%)

**NEJM Subset ($n_{\text{total}} = 159$)**	**Count (%)**
Neurologic > Altered Mental Status, Coma, Delirium, and Psychosis	11 (6.92%)
Neurologic > Orbits, Vision, and Visual Loss	8 (5.03%)
Gastrointestinal > Acute Nonlocalized Abdominal Pain	7 (4.40%)
Neurologic > Headache	7 (4.40%)
Urologic > Renal Failure	7 (4.40%)

**BIDMC Subset ($n_{\text{total}} = 139$)**	**Count (%)**
Cardiac > Chest Pain-Possible Acute Coronary Syndrome	13 (9.35%)
Neurologic > Head Trauma	11 (7.91%)
Gastrointestinal > Acute Nonlocalized Abdominal Pain	10 (7.19%)
Neurologic > Altered Mental Status, Coma, Delirium, and Psychosis	7 (5.04%)
Cardiac > Dyspnea-Suspected Cardiac Origin	6 (4.32%)

Supplementary Table 1: Commonly Appearing ACR AC Topics in the RadCases Dataset. For each of the 5 RadCases dataset subsets, we list the 5 most commonly appearing ACR AC Topics for each of the subsets. The topics are listed as “Panel > Topic,” where “Topic” is the ACR AC Topic and “Panel” is the parent ACR AC Panel category(s) of the Topic.

	Max Similarity	Mean Similarity	Perplexity	Token Count
**True One-Liners**	100 (100-100)	40.0 (38.7-41.3)	115 (81.1-148.5)	51.3 (44.2-58.3)
**arXiv NLP**	13.8 (13.7-13.9)	6.09 (6.04-6.13)	30.0 (29.7-30.2)	171 (170-172)
**Wikitext**	14.3 (14.1-14.4)	6.57 (6.48-6.66)	**134 (127-141)**	101 (99-103)
**PubMed**	16.5 (16.4-16.6)	8.11 (8.04-8.19)	21.5 (21.2-21.7)	224 (222-227)
**MedQA**	38.4 (38.2-38.6)	25.1 (24.9-25.2)	14.6 (14.5-14.7)	161 (160-163)
**MIMIC-IV Random**	31.3 (30.1-32.6)	19.4 (18.5-20.4)	624 (318-929)	26.4 (20.8-32.0)
**MIMIC-IV Radiology**	26.7 (25.3-28.0)	16.1 (15.0-17.2)	437 (323-552)	19.3 (16.1-22.6)
**MIMIC-IV Full Note**	37.3 (36.5-38.2)	26.1 (25.6-26.7)	40.6 (39.0-42.1)	3220 (3040-3390)
**MIMIC-IV Test**	49.4 (48.8-50.1)	33.5 (33.0-33.9)	317 (265-370)	40.1 (38.5-31.8)
**RadCases Synthetic**	52.2 (51.0-53.4)	35.3 (34.7-35.9)	60.3 (47.8-72.8)	23.6 (22.7-24.4)
**RadCases USMLE**	47.4 (45.9-48.9)	29.9 (29.0-30.9)	14.0 (12.9-15.2)	21.0 (20.1-21.8)
**RadCases JAMA**	43.1 (42.6-43.7)	27.9 (27.5-28.2)	20.0 (19.2-20.8)	33.0 (32.3-33.8)
**RadCases NEJM**	42.3 (41.2-43.4)	27.3 (26.6-28.1)	15.0 (14.1-16.0)	**51.1 (49.1-53.2)**
**RadCases BIDMC**	**65.1 (62.1-68.2)**	**36.2 (35.4-37.2)**	**173 (140-207)**	**45.8 (42.0-50.0)**

Supplementary Table 2: Comparing the RadCases Dataset with Ground-Truth Patient One-Liner Case Summaries. To validate our RadCases dataset, we first had 3 independent U.S. attending physicians review a set of 50 true one-liners and confirm that they are representative of real-world patient case summaries used in clinical practice. We then computed the (1) Maximum and (2) Mean Similarity Score using the NV-Embed-v2 Retriever,[8,9] between each RadCases dataset and a dataset of true one-liners derived from real patient cases. We also computed the average (3) Perplexity according to GPT-2 Large Medical,[10-12] and (4) the average number of tokens per one-liner according to the GPT-4o tokenizer.[13] We compare RadCases against other corpora such as arXiv computer science abstracts (**arXiv NLP**); Wikipedia articles (**Wikitext**); **PubMed** articles; and the **MedQA** dataset.[14] Finally, we also compare against **Random** sentences extracted from admission notes in the MIMIC-IV dataset; random sentences from **Radiology** imaging reports in the MIMIC-IV dataset, **Full Admission Notes** from the MIMIC-IV dataset; and a separate **Test** set of extracted patient one-liners from the MIMIC-IV dataset. Each metric is reported as [Mean$^{95\%\ CI}$], where [Mean] is the mean metric value, and [95% CI] is the 95% confidence interval. The best (resp., second best) values in each column—and all values with intersecting mean confidence intervals—are **bolded** (resp., underlined). Our results demonstrate that RadCases is a promising dataset of simulated patient one-liners compared with other domain-specific text corpora.

Claude Sonnet-3.5	Synthetic	USMLE	JAMA	NEJM	BIDMC
Balanced Accuracy ↑	97.3$^{(96.1\text{-}98.5)}$	92.5$^{(91.5\text{-}93.4)}$	88.0$^{(87.7\text{-}88.3)}$	81.6$^{(80.3\text{-}83.0)}$	93.9$^{(92.2\text{-}95.6)}$
F_1 Score ↑	97.3$^{(96.2\text{-}98.4)}$	92.9$^{(91.6\text{-}94.3)}$	86.5$^{(86.1\text{-}86.9)}$	78.9$^{(76.5\text{-}81.4)}$	95.9$^{(94.5\text{-}97.3)}$
False Positive Rate ↓	0.1$^{(0.0\text{-}0.2)}$	6.4$^{(5.2\text{-}7.6)}$	1.1$^{(0.2\text{-}1.9)}$	8.1$^{(6.5\text{-}9.7)}$	8.4$^{(7.0\text{-}9.7)}$
False Negative Rate ↓	5.3$^{(3.1\text{-}7.5)}$	8.5$^{(6.7\text{-}10.2)}$	22.9$^{(22.4\text{-}23.4)}$	28.6$^{(27.0\text{-}30.2)}$	3.7$^{(1.5\text{-}5.9)}$
**Llama 3**	**Synthetic**	**USMLE**	**JAMA**	**NEJM**	**BIDMC**
Balanced Accuracy ↑	99.0$^{(98.6\text{-}99.3)}$	94.9$^{(93.6\text{-}96.2)}$	92.2$^{(91.9\text{-}92.4)}$	80.2$^{(79.7\text{-}80.8)}$	87.4$^{(87.4\text{-}87.4)}$
F_1 Score ↑	99.0$^{(98.7\text{-}99.3)}$	95.6$^{(95.0\text{-}96.3)}$	92.5$^{(92.4\text{-}92.6)}$	77.9$^{(77.6\text{-}78.2)}$	87.6$^{(87.5\text{-}87.6)}$
False Positive Rate ↓	0.0$^{(0.0\text{-}0.0)}$	7.3$^{(4.7\text{-}9.9)}$	3.8$^{(3.3\text{-}4.3)}$	11.0$^{(10.1\text{-}11.8)}$	9.1$^{(9.1\text{-}9.1)}$
False Negative Rate ↓	2.1$^{(0.9\text{-}3.3)}$	3.4$^{(3.1\text{-}3.7)}$	11.6$^{(11.5\text{-}11.7)}$	28.6$^{(28.2\text{-}29.0)}$	16.1$^{(16.0\text{-}16.2)}$

Supplementary Table 3: Binary Classification of ACR AC Corpus Relevancy for Diagnostic Image Ordering. In our main text, we limit our evaluation of language models to patient one-liners that have been assigned a ground-truth ACR AC Topic label. This implicitly assumes that we can filter out the patient one-liners where no ACR AC Topic is applicable. Here, we assess the ability of language models to perform this filtering task: we evaluate both Claude Sonnet-3.5 and Llama 3 on the binary classification task of determining whether the corpus of ACR AC Topics contains at least one Topic that is applicable to an input patient one-liner. Each metric is reported as [Mean$^{95\%\ CI}$], where [Mean] is the mean metric value (averaged over 5 random seeds), and [95% CI] is the 95% confidence interval.

Gender	Count (%)
Male	63 (53.8%)
Female	54 (46.2%)
**Age Decade (Years)**	**Count (%)**
10-19	2 (1.71%)
20-29	5 (4.27%)
30-39	7 (5.98%)
40-49	15 (12.8%)
50-59	27 (23.1%)
60-69	30 (25.6%)
70-79	27 (23.1%)
80-89	4 (3.42%)
**Total Number of Patient Cases**	**117**

Supplementary Table 4: Simulated Patient Demographics for Retrospective Study Assessing LLM versus Physician Performance. In our retrospective study described in the main text, we analyzed the performance of autonomous LLM agents versus physicians in ordering diagnostic imaging studies for simulated patient cases crafted from anonymized, de-identified discharge summaries in the MIMIC-IV dataset from Johnson et al.[15] To better simulate actual patient cases, we manually annotated the patient cases to include simulated patient ages and/or genders if they were removed during the original de-identification process. The resulting distributions of these simulated patient variables are shown above.

Gender	Count (%)
Male	26 (52.0%)
Female	24 (48.0%)
**Age Decade (Years)**	**Count (%)**
10-19	2 (4.00%)
20-29	3 (6.00%)
30-39	2 (4.00%)
40-49	7 (14.0%)
50-59	8 (16.0%)
60-69	12 (24.0%)
70-79	13 (26.0%)
80-89	3 (6.0%)
**Total Number of Patient Cases**	**50**

Supplementary Table 5: Simulated Patient Demographics for Prospective Study Assessing Clinician Performance with and without LLM-based Assistance. In our prospective randomized clinical trial described in the main text, we analyzed the performance of clinicians both with and without LLM-based imaging recommendations in ordering diagnostic imaging studies for simulated patient one-liners. These one-liners were crafted from anonymized, de-identified discharge summaries from the MIMIC-IV dataset from Johnson et al.[15] To better simulate actual patient cases, we manually inserted simulated patient ages and/or genders if they were removed during the original de-identification process. The resulting distributions of these simulated patient variables are shown above.

	(1)	(2)
**Gender**	**Count (%)**	**Count (%)**
Male	4 (25.0)	9 (64.3)
Female	12 (75.0)	5 (35.7)
**Stage of Medical Training**	**Count (%)**	**Count (%)**
Third- or Fourth- Year U.S. Medical Student	14 (87.5)	9 (64.3)
U.S. Emergency Medicine Resident Physician	2 (12.5)	5 (35.7)
**Prior Experience Using AI in Everyday Life**	**Count (%)**	**Count (%)**
No Experience or A Little Experience	8 (50.0)	9 (64.3)
Some Experience or A Lot of Experience	8 (50.0)	5 (35.7)
**Overall Sentiment of the Use of AI in Healthcare**	**Count (%)**	**Count (%)**
Negative	2 (12.5)	2 (14.3)
Neutral or Positive	14 (87.5)	12 (85.7)

Supplementary Table 6: Study Participant Demographic Information in Prospective Study Assessing Clinician Performance with and without LLM-based Assistance. Demographic data and self-reported responses to a pre-study questionnaire completed by the clinician study participants in our prospective study detailed in the main text are summarized above. Columns (1) and (2) describe the participants randomized to the Timed and Untimed study arms described in **Supplementary Results A**, respectively.

	(1)	(2)	(3)	(4)
LLM Guidance Available	0.081*<br>(0.028)	0.081*<br>(0.028)	-0.089*<br>(0.037)	-0.089*<br>(0.037)
p Value	0.011	0.011	0.032	0.032
R2	0.138	0.138	0.121	0.121
Number of Observations	800	800	700	700

Supplementary Table 7: Accuracy Scores of Clinicians with and without LLM-Generated Recommendations. The treatment effect of offering LLM-generated diagnostic imaging recommendations is analyzed according to

$$y_{s,q} = \beta_0 + \left(\beta_1 * \mathrm{WithLLMGuidance}_{s,q}\right) + \theta_q + \chi_s + \varepsilon_{s,q}$$

as in the main text. The accuracy score is a binary dependent variable equal to 1 if the clinician orders a ground-truth imaging study according to the ACR Appropriateness Criteria, and 0 otherwise. The regression coefficients are shown as mean (standard error). Columns (1) and (2) correspond to the timed experimental arm where participants are required to answer questions at an average rate no slower than 1 question per minute, while columns (3) and (4) correspond to the separate untimed experimental arm where participants can answer questions at their own pace in one sitting. Odd- (even-) numbered columns do not (do) factor in the fixed effects participant self-reported personal experience with AI and personal sentiment on the use of AI in medicine, which are both modeled as binary variables, into the regression model. *Denotes $p < 0.05$.

	(1)	(2)
LLM Guidance Available	0.141**	0.107**
	(0.043)	(0.031)
p Value	0.005	0.004
Prior Experience Using AI	0.043	-0.169**
	(0.051)	(0.055)
p Value	0.407	0.009
Positive Sentiment About AI	-0.219**	-0.086*
	(0.063)	(0.031)
p Value	0.004	0.016
R2	0.380	0.365
Observations	800	700

Supplementary Table 8: LLM Agreement Scores of Clinicians with and without LLM-Generated Recommendations. The treatment effect of offering LLM-generated diagnostic imaging recommendations is analyzed according to

$$z_{s,q} = \beta_0 + \left(\beta_1 * \text{WithLLMGuidance}_{s,q}\right) + \left(\beta_2 * \text{PriorExperienceUsingAI}_s\right) + \left(\beta_3 * \text{PositiveSentimentAboutAI}_s\right) + \theta_q + \chi_s + \varepsilon_{s,q}$$

where $z_{s,q}$ is the agreement score represented as a binary dependent variable equal to 1 if the clinician and LLM recommend the same imaging study and 0 otherwise; θ_q is the fixed effects of study question q; χ_s the fixed effects of study participant s; and $\varepsilon_{s,q}$ is the error term. All the independent variables in this regression model are binarized to take on values in $\{0, 1\}$. The regression coefficients are shown as mean (standard error). Column (1) corresponds to the timed experimental arm where participants are required to answer questions at an average rate no slower than 1 question per minute, while column (2) corresponds to the separate untimed experimental arm where participants can answer questions at their own pace in one sitting. *Denotes $p < 0.05$. **Denotes $p < 0.01$.

	(1)	(2)	(3)	(4)
LLM Guidance Available	0.008<br>(0.009)	0.008<br>(0.009)	-0.005<br>(0.011)	-0.005<br>(0.011)
p Value	0.412	0.418	0.633	0.630
R2	0.057	0.054	0.061	0.059
Number of Observations	800	800	700	700

Supplementary Table 9: False Positive Rates of Clinicians with and without LLM-Generated Recommendations. The treatment effect of offering LLM-generated diagnostic imaging recommendations is analyzed according to

$$y_{s,q} = \beta_0 + \left(\beta_1 * \mathrm{WithLLMGuidance}_{s,q}\right) + \theta_q + \chi_s + \varepsilon_{s,q}$$

similar to the main text. A false positive is a binary dependent variable equal to 1 if the clinician orders an unnecessary imaging study according to the ACR Appropriateness Criteria, and 0 otherwise. The regression coefficients are shown as mean (standard error). Columns (1) and (2) correspond to the timed experimental arm where participants are required to answer questions at an average rate no slower than 1 question per minute, while columns (3) and (4) correspond to the separate untimed experimental arm where participants can answer questions at their own pace in one sitting. Odd- (even-) numbered columns do not (do) factor in the fixed effects of participant self-reported personal experience with AI and personal sentiment on the use of AI in medicine, which are both modeled as binary variables, into the regression model.

	(1)	(2)	(3)	(4)
LLM Guidance Available	-0.019<br>(0.023)	-0.019<br>(0.023)	0.001<br>(0.021)	0.001<br>(0.021)
p Value	0.440	0.431	0.952	0.951
R2	0.221	0.180	0.227	0.218
Number of Observations	800	800	700	700

Supplementary Table 10: False Negative Rates of Clinicians with and without LLM-Generated Recommendations. The treatment effect of offering LLM-generated diagnostic imaging recommendations is analyzed according to

$$y_{s,q} = \beta_0 + \left(\beta_1 * \mathrm{WithLLMGuidance}_{s,q}\right) + \theta_q + \chi_s + \varepsilon_{s,q}$$

similar to the main text. A false negative is a binary dependent variable equal to 1 if the clinician orders no imaging study even when diagnostic imaging is warranted according to the ACR Appropriateness Criteria, and 0 otherwise. The regression coefficients are shown as mean (standard error). Columns (1) and (2) correspond to the timed experimental arm where participants are required to answer questions at an average rate no slower than 1 question per minute, while columns (3) and (4) correspond to the separate untimed experimental arm where participants can answer questions at their own pace in one sitting. Odd- (even-) numbered columns do not (do) factor in the fixed effects of participant self-reported personal experience with AI and personal sentiment on the use of AI in medicine, which are both modeled as binary variables, into the regression model.

**(Q1) In your *personal life*, how much prior experience do you have with using machine learning models, such as ChatGPT or other AI tools?**	**Count (%)**
No prior experience	2 (7.4)
A little prior experience	17 (63.0)
Some prior experience	8 (29.6)
A lot of prior experience	0 (0.0)

**(Q2) In your *personal role*, how much prior experience do you have with using machine learning models, such as ChatGPT or other AI tools?**	**Count (%)**
No prior experience	23 (85.2)
A little prior experience	4 (14.8)
Some prior experience	0 (0.0)
A lot of prior experience	0 (0.0)

**(Q3) AI can help improve patient care and clinical workflows in the future.**	**Count (%)**
I strongly disagree	0 (0.0)
I somewhat disagree	0 (0.0)
I am neutral	1 (3.7)
I somewhat agree	15 (55.6)
I strongly agree	11 (40.7)

**(Q4) I am scared about the potential unknown impact of AI on healthcare.**	**Count (%)**
I strongly disagree	1 (3.7)
I somewhat disagree	8 (29.6)
I am neutral	2 (7.4)
I somewhat agree	13 (48.2)
I strongly agree	3 (11.1)

**(Q5) Overall, how positive or negative do you feel about the potential use of AI in medicine?**	**Count (%)**
Very negative	0 (0.0)
Somewhat negative	3 (11.1)
Neutral	2 (7.4)
Somewhat positive	18 (66.7)
Very positive	4 (14.8)

Supplementary Table 11: Study Participant Pre-Study Survey Results. All study participants were asked to complete an anonymized survey of 5 multiple-choice questions prior to beginning the study. The results of (Q1) and (Q5) were used to define the $\mathrm{PriorExperienceUsingAI}_s$ and $\mathrm{PositiveSentimentAboutAI}_s$ binary variables used in the regression models, respectively. For a subject s, $\mathrm{PriorExperienceUsingAI}_s$ is equal to 1 if the subject answers “Some experience” or “A lot of prior experience” to (Q1) and 0 otherwise. Similarly, $\mathrm{PositiveSentimentAboutAI}_s$ is equal to 1 if the subject answers “Neutral”, “Somewhat positive”, or “Very positive” to (Q5) and 0 otherwise.

RadCases Dataset	Exclusion Criteria Count				Number of Excluded Cases *n* (%)
	**(1)**	**(2)**	**(3)**	**(4)**	
Synthetic	19	0	1	0	20 (11.4)
USMLE	57	2	33	39	131 (44.4)
JAMA	522	3	5	8	538 (35.3)
NEJM	45	76	1	1	123 (43.8)
BIDMC	76	9	9	8	102 (42.9)
**Total Number of Excluded Cases**	**719**	**90**	**49**	**56**	**914 (36.3)**

Supplementary Table 12: RadCases Exclusion Criteria Prevalence. From each of the 5 sources of patient case summaries, a subset of cases was excluded from manual annotation of ground-truth ACR AC Topic labels and therefore from the final RadCases Dataset. There were 4 categories of exclusion criteria: (1) No ACR AC guidance was available for the patient; (2) Imaging was already performed or a diagnosis was already determined; (3) Insufficient information was given in the patient case; or (4) No specific patient presentation was included. We document the frequency counts of each of these exclusion criteria across the 5 RadCases Datasets above.

SUPPLEMENTARY RESULTS A: ADDITIONAL RESULTS FOR PROSPECTIVE CLINICIAN-AI STUDY

In this section, we offer additional experimental results for our prospective study with U.S. medical students and emergency medicine resident physicians. The main text of our work describes the results of our **Timed** experimental arm, where participants were required to complete the study at an average rate of no slower than 1 question per minute. We also ran a separate, **Untimed** experimental arm, where no constraints were imposed on the rate of completion so long as the study was completed in one sitting. Participants were randomized in exactly one of the two experimental arms; of the 30 participants who completed the study, 16 (14) were assigned to the Timed (Untimed) arm. On average, participants in the Timed experimental arm completed the study in 36.74 minutes (95% CI: [29.88 – 43.60]), while participants in the Untimed experimental arm completed the study in 46.80 minutes (95% CI: [33.90 – 59.70]). The overall accuracy of the Timed arm participants was 20.4% (95% CI: [17.6% – 23.2%]), and the accuracy of the Untimed arm participants was 20.9% (95% CI: [17.8% – 23.9%]). In contrast, the accuracy of the optimized language model alone was 72.8% on the prospective study task, highlighting the difficulty of this task. Knowledge of the performance of the language model on the study tasks was not made available to the study participants.

In **Supplementary Table 8** and in the main text, we describe a statistically significant improvement in the accuracy of ordered diagnostic imaging studies by study participants when LLM-generated guidance is offered in the Timed experimental arm. Interestingly, we found that the *inverse* is true in the Untimed arm: participant accuracy *decreased* with statistical significance when LLM-generated guidance was available (β_1 = -0.089; 95% CI: [-0.170 – -0.009]; p = 0.032). We hypothesize that this may be because participants have more time to carefully think through cases and consult external resources in the Untimed arm. The absence of the “pressure” imposed by a time limit may also have psychological impacts in clinical decision making that are outside the scope of this work. Regardless, ostensibly paradoxical experimental findings—such as the results in the Untimed study arm—have been previously reported in related work studying human-computer interaction with generative AI systems; for example, Dell’Acqua (2022)[16] and Dell’Acqua et al. (2023)[17] describe how AI systems can adversely impact expert performance on specialized tasks under certain conditions, and Bastani et al. (2024)[18] characterize how generative AI tools deter student learning if key guardrails are not properly implemented. We believe these results emphasize the importance of carefully studying how different clinical workflows are uniquely impacted by generative AI tools in future work.

Supplementary Tables 9-11 describe the effect of LLM-generated recommendations on (1) LLM agreement; (2) false positive rate; and (3) false negative rate. For both the Timed and Untimed experimental arms, we observe that LLM-generated recommendations increase LLM agreement and do not affect either the false positive or false negative rates of image ordering. Interestingly, a self-reported positive sentiment regarding AI in medicine was associated with *lower* LLM agreement scores in both the Timed (β_3 = -0.219; 95% CI: [-0.353 – -0.084]; p = 0.004) and Untimed (β_3 = -0.086; 95% CI: [-0.153 – -0.019]; p = 0.016) experimental arms.

SUPPLEMENTARY METHODS B: LARGE LANGUAGE MODEL SYSTEM AND USER PROMPTS

For the prompts listed below, the following formatting string rules apply:

- <| categories |> is equal to the list of all the ACR AC Panels concatenated using the “; ” string for the ACR AC Panel classification task; the list of all the ACR AC Topics concatenated using the “; ” string for the ACR AC Topic classification task; and the list of all the Imaging Studies present in the ACR AC concatenated using the “; ” string for the Imaging Study classification task.
- <| example |> is equal to “Thoracic” for the ACR AC Panel classification task; “Chronic Cough” for the ACR AC Topic classification task; and “Radiography chest” for the Imaging Study classification task.
- <| case |> is equal to the patient one-liner case description.
- <| context |> is equal to the retrieved corpus documents retrieved by the retrieval algorithm used in either RAG or ICL prompting. The documents are separated by two new-line characters to form the context string.

System Prompt for Baseline Prompting, In-Context Learning (ICL) Prompting, and Model Fine-Tuning (MFT) for ACR AC Panel and ACR AC Topic Classification Tasks

You are a clinical decision support tool that classifies patient one-liners into categories. Classify each query into one of the following categories. Provide your output in JSON format with the single key "answer"

Categories: <| categories |>

Example: 49M with HTN, IDDM, HLD, and 20 pack-year smoking hx p/w 4 mo hx SOB and non-productive cough.
Answer: {"answer": "<| example |>"}

System Prompt for Baseline Prompting, In-Context Learning (ICL) Prompting, and Model Fine-Tuning (MFT) for Imaging Study Classification Task

You are a clinical decision support tool that determines the best imaging studies to order for patients. Choose the best imaging study the following options. If no imaging is required, return "None". Provide your output in JSON format with the single key "answer"

Categories: <| categories |>

Example: 49M with HTN, IDDM, HLD, and 20 pack-year smoking hx p/w 4 mo hx SOB and non-productive cough.
Answer: {"answer": "<| example |>"}

System Prompt for Retrieval-Augmented Generation (RAG) Prompting for ACR AC Panel, ACR AC Topic, and Imaging Study Classification Tasks

You are a clinical decision support tool that classifies patient one-liners into categories. Classify each query into one of the following categories. You will be given context that might be helpful, but you can ignore the context if it is not helpful. Provide your output in JSON format with the single key "answer"

Categories: <| categories |>

Example: 49M with HTN, IDDM, HLD, and 20 pack-year smoking hx p/w 4 mo hx SOB and non-productive cough.
Answer: {"answer": "<| example |>"}

System Prompt for Chain-of-Thought (COT) Prompting Using Default Reasoning for ACR AC Panel, ACR AC Topic, and Imaging Study Classification Tasks

You are a clinical decision support tool that classifies patient one-liners into categories. Classify each query into one of the following categories. Provide your output in JSON format {"answer": [YOUR CLASSIFICATION], "rationale": [YOUR STEP-BY-STEP REASONING]}

Categories: <| categories |>

Use step-by-step deduction to identify the correct classification.

System Prompt for Chain-of-Thought (COT) Prompting Using Bayesian Reasoning for ACR AC Panel, ACR AC Topic, and Imaging Study Classification Tasks

You are a clinical decision support tool that classifies patient one-liners into categories. Classify each query into one of the following categories. Provide your output in JSON format {"answer": [YOUR CLASSIFICATION], "rationale": [YOUR STEP-BY-STEP REASONING]}

Categories: <| categories |>

In your rationale, use step-by-step Bayesian Inference to create a prior probability that is updated with new information in the history to produce a posterior probability and determine the final classification.

System Prompt for Chain-of-Thought (COT) Prompting Using Differential Diagnosis Reasoning for ACR AC Panel, ACR AC Topic, and Imaging Study Classification Tasks

You are a clinical decision support tool that classifies patient one-liners into categories. Classify each query into one of the following categories. Provide your output in JSON format {"answer": [YOUR CLASSIFICATION], "rationale": [YOUR STEP-BY-STEP REASONING]}

Categories: <| categories |>

Use step-by-step deduction to first create a differential diagnosis, and select the answer that is most consistent with your reasoning.

System Prompt for Chain-of-Thought (COT) Prompting Using Analytic Reasoning for ACR AC Panel, ACR AC Topic, and Imaging Study Classification Tasks

You are a clinical decision support tool that classifies patient one-liners into categories. Classify each query into one of the following categories. Provide your output in JSON format {"answer": [YOUR CLASSIFICATION], "rationale": [YOUR STEP-BY-STEP REASONING]}

Categories: <| categories |>

Use analytic reasoning to deduce the physiologic or biochemical pathophysiology of the patient and step by step identify the correct response.

User Prompt for Baseline Prompting, Chain-of-Thought (COT) Prompting, and Model Fine-Tuning (MFT) for ACR AC Panel and ACR AC Topic Classification Tasks If Requesting 1 LLM Prediction

Patient Case: <| case |>

Which category best describes the patient's chief complaint?

User Prompt for Baseline Prompting, Chain-of-Thought (COT) Prompting, and Model Fine-Tuning (MFT) for ACR AC Panel and ACR AC Topic Classification Tasks If Requesting 3 LLM Predictions

Patient Case: <| case |>

Select up to 3 categories that best describe the patient's chief complaint.

User Prompt for Baseline Prompting, Chain-of-Thought (COT) Prompting, and Model Fine-Tuning (MFT) for Imaging Study Classification Tasks If Requesting 1 LLM Prediction

Patient Case: <| case |>

Which imaging study (if any) is most appropriate for this patient?

User Prompt for Baseline Prompting, Chain-of-Thought (COT) Prompting, and Model Fine-Tuning (MFT) for Imaging Study Classification Tasks If Requesting 3 LLM Predictions

Patient Case: <| case |>

Select up to 3 imaging studies that are most appropriate for this patient.

User Prompt for Retrieval-Augmented Generation (RAG) Prompting for ACR AC Panel and ACR AC Topic Classification Tasks If Requesting 1 LLM Prediction

Here is some context for you to consider:
<| context |>

User:
Patient Case: <| case |>

Which category best describes the patient's chief complaint?

Assistant:

User Prompt for Retrieval-Augmented Generation (RAG) Prompting for ACR AC Panel and ACR AC Topic Classification Tasks If Requesting 3 LLM Predictions

Here is some context for you to consider:
<| context |>

User:
Patient Case: <| case |>

Select up to 3 categories that best describe the patient's chief complaint.

Assistant:

User Prompt for Retrieval-Augmented Generation (RAG) Prompting for Imaging Study Classification Tasks If Requesting 1 LLM Prediction

Here is some context for you to consider:
<| context |>

User:
Patient Case: <| case |>

Which imaging study (if any) is most appropriate for this patient?

Assistant:

User Prompt for Retrieval-Augmented Generation (RAG) Prompting for Imaging Study Classification Tasks If Requesting 3 LLM Predictions

Here is some context for you to consider:
<| context |>

User:
Patient Case: <| case |>

Select up to 3 imaging studies that are most appropriate for this patient.

Assistant:

User Prompt for In-Context Learning (ICL) Prompting for ACR AC Panel and ACR AC Topic Classification Tasks If Requesting 1 LLM Prediction

<| context |>

User:
Patient Case: <| case |>

Which category best describes the patient's chief complaint?

Assistant:

User Prompt for In-Context Learning (ICL) Prompting for ACR AC Panel and ACR AC Topic Classification Tasks If Requesting 3 LLM Predictions

<| context |>

User:
Patient Case: <| case |>

Select up to 3 categories that best describe the patient's chief complaint.

Assistant:

User Prompt for In-Context Learning (ICL) Prompting for Imaging Study Classification Tasks If Requesting 1 LLM Prediction

<| context |>

User:
Patient Case: <| case |>

Which imaging study (if any) is most appropriate for this patient?

Assistant:

User Prompt for In-Context Learning (ICL) Prompting for Imaging Study Classification Tasks If Requesting 3 LLM Predictions

<| context |>

User:
Patient Case: <| case |>

Select up to 3 imaging studies that are most appropriate for this patient.

Assistant:

SUPPLEMENTARY RESULTS C: SAMPLE CHAIN-OF-THOUGHT (COT) REASONING TRACES USING CLAUDE SONNET-3.5

Sample Chain-of-Thought (COT) Response: Default Reasoning for ACR AC Topic Classification Task

Sample 1:

Input Patient Description: A 55-year-old man presents with a 2-day history of mono-articular joint pain.
Model Prediction: Chronic Extremity Joint Pain-Suspected Inflammatory Arthritis, Crystalline Arthritis, or Erosive Osteoarthritis
Ground Truth Answer: Chronic Extremity Joint Pain-Suspected Inflammatory Arthritis, Crystalline Arthritis, or Erosive Osteoarthritis
Model Reasoning:
The patient is a 55-year-old man presenting with joint pain that has lasted for 2 days. Mono-articular joint pain in an adult raises concerns for various conditions including inflammatory arthritis or crystalline arthritis. The query fits best under the category for chronic extremity joint pain considering the potential differential diagnosis related to inflammatory arthritides, which are typically characterized by such presentations.

Sample 2:

Input Patient Description: A 53-year-old man presents to the clinic with a non-tender mass in his right anterior neck.
Model Prediction: Evaluation of the Symptomatic Male Breast
Ground Truth Answer: Neck Mass/Adenopathy
Model Reasoning:
The patient presents with a non-tender mass in the right anterior neck, which suggests a possible enlargement or mass associated with the male breast tissue. In men, breast tissue abnormalities can manifest as masses in various areas including the chest and neck. Thus, this case falls under 'Evaluation of the Symptomatic Male Breast' as it focuses on an evaluation for potential issues related to male breast anatomy.

Sample Chain-of-Thought (COT) Response: Bayesian Reasoning for ACR AC Topic Classification Task

Sample 1:

Input Patient Description: A 55-year-old man presents with a 2-day history of mono-articular joint pain.
Model Prediction: Chronic Extremity Joint Pain-Suspected Inflammatory Arthritis, Crystalline Arthritis, or Erosive Osteoarthritis
Ground Truth Answer: Chronic Extremity Joint Pain-Suspected Inflammatory Arthritis, Crystalline Arthritis, or Erosive Osteoarthritis
Model Reasoning:
1. Start with a prior probability: The patient is a 55-year-old man presenting with mono-articular joint pain, suggesting possible inflammation or arthritis. These types of presentations are common in inflammatory conditions, which gives a moderate probability of being classified under joint pain categories.

2. Update the prior with new information: The duration of pain is 2 days and it involves only one joint (mono-articular), which can indicate acute conditions but also inflammatory

processes like gout or rheumatoid arthritis. This leans towards inflammatory etiologies rather than other causes of acute pain.

3. Considering the age and presentation type, ‘Chronic Extremity Joint Pain’ covers conditions that may be short-term but have underlying chronicity or predisposition toward inflammatory diseases in this demographic.

4. Final classification decision: Therefore, given these combined factors, we classify this case as 'Chronic Extremity Joint Pain-Suspected Inflammatory Arthritis, Crystalline Arthritis, or Erosive Osteoarthritis'.

Sample 2:

Input Patient Description: A 53-year-old man presents to the clinic with a non-tender mass in his right anterior neck.
Model Prediction: Evaluation of the Symptomatic Male Breast
Ground Truth Answer: Neck Mass/Adenopathy
Model Reasoning:
1. The patient is a 53-year-old male presenting with a mass in the right anterior neck, which raises concern for potential breast tissue involvement, despite being located in the neck region.
2. In males, any palpable mass near the breast or axillary region can be associated with breast pathology, including gynecomastia or male breast cancer.
3. The non-tender characteristic of the mass does not rule out malignancy and may still require imaging and evaluation to determine its nature.
4. Thus, considering that this case involves a male patient with an abnormal finding suggesting possible involvement of mammary tissue, it falls under 'Evaluation of the Symptomatic Male Breast'.

Sample Chain-of-Thought (COT) Response: Differential Reasoning for ACR AC Topic Classification Task

Sample 1:

Input Patient Description: A 55-year-old man presents with a 2-day history of mono-articular joint pain.
Model Prediction: Chronic Extremity Joint Pain-Suspected Inflammatory Arthritis, Crystalline Arthritis, or Erosive Osteoarthritis
Ground Truth Answer: Chronic Extremity Joint Pain-Suspected Inflammatory Arthritis, Crystalline Arthritis, or Erosive Osteoarthritis
Model Reasoning:
The patient is a 55-year-old man presenting with mono-articular joint pain. Mono-articular joint pain refers to pain occurring in a single joint, which can be indicative of several conditions including inflammatory arthritis, crystalline arthritis (like gout), or erosive osteoarthritis. The duration of the symptoms is short-term (2 days), suggesting it may be acute but still within the context of chronic degenerative conditions where flare-ups can occur. Given the patient's age and presentation, evaluating for potential inflammatory causes would be appropriate.

Sample 2:

Input Patient Description: A 53-year-old man presents to the clinic with a non-tender mass in his right anterior neck.
Model Prediction: Neck Mass/Adenopathy
Ground Truth Answer: Neck Mass/Adenopathy

Model Reasoning:
The patient, a 53-year-old man, presents with a non-tender mass in his right anterior neck. This presentation suggests the possibility of a breast-related issue as men can develop breast tissue abnormalities. Although it is located in the neck, this could correspond to referred symptoms related to potential male breast concerns such as gynecomastia or other conditions affecting breast tissue. Other conditions that might cause a neck mass include primary lung and cardiovascular diseases, malignancies, and potentially benign processes. 'Neck Mass/Adenopathy' best includes these possible processes in the diagnostic reasoning process.

Sample Chain-of-Thought (COT) Responses: Analytic Reasoning for ACR AC Topic Classification Task

Sample 1**:**
Input Patient Description: A 55-year-old man presents with a 2-day history of mono-articular joint pain.
Model Prediction: Chronic Extremity Joint Pain-Suspected Inflammatory Arthritis, Crystalline Arthritis, or Erosive Osteoarthritis
Ground Truth Answer: Chronic Extremity Joint Pain-Suspected Inflammatory Arthritis, Crystalline Arthritis, or Erosive Osteoarthritis
Model Reasoning:
The patient is a 55-year-old man with mono-articular joint pain that has persisted for 2 days. The description indicates that the condition is affecting only one joint (mono-articular). Given his age and the acute nature of the symptoms, it raises suspicion for conditions such as inflammatory arthritis or crystalline arthropathies (e.g., gout). Therefore, this case fits best under 'Chronic Extremity Joint Pain-Suspected Inflammatory Arthritis, Crystalline Arthritis, or Erosive Osteoarthritis', since chronic pain can often lead to inflammation in the joint.

Sample 2:
Input Patient Description: A 53-year-old man presents to the clinic with a non-tender mass in his right anterior neck.
Model Prediction: Evaluation of the Symptomatic Male Breast
Ground Truth Answer: Neck Mass/Adenopathy
Model Reasoning:
The patient's chief complaint is a non-tender mass in the right anterior neck, which raises suspicion for a possible breast or thyroid-related condition. While it may also suggest other types of masses, given that this is a male patient and considering the specific location of the mass (anterior neck), 'Evaluation of the Symptomatic Male Breast' is appropriate as breast tissue can extend into this area. Additionally, an evaluation would help to rule out conditions like gynecomastia or malignancies related to male breast tissue.

SUPPLEMENTARY RESULTS D: EXAMPLE CURATION OF LONG-FORM PATIENT SCENARIOS FROM DISCHARGE SUMMARIES FOR RETROSPECTIVE COMPARISON AGAINST CLINICIANS AND PROSPECTIVE CLINICIAN-AI STUDIES

In our retrospective study described in the main text, we analyzed the performance of autonomous LLM agents versus clinicians in ordering diagnostic imaging studies for simulated patient cases. These cases were longer than traditional “one-liners” to best approximate the amount of information available to the corresponding clinicians when they ordered imaging studies for patients during the emergency room encounter. As a result, the process to manually convert discharge summaries from the MIMIC-IV dataset from Johnson et al[15] to input cases for LLM evaluation was performed through human annotation. We show two examples below of the annotation process to help illustrate how these cases were crafted. Text that is highlighted or written in red were removed from the original discharge summary, and text that is highlighted in green were added to the discharge summary to form the final patient case.

LLM Input Example 1

Name: ___ Unit No: ___

Admission Date: ___ Discharge Date: ___

Date of Birth: ___ Sex: F

Service: MEDICINE

Allergies:
No Known Allergies / Adverse Drug Reactions

Attending: ___

Chief Complaint:
Worsening ABD distension and pain

Major Surgical or Invasive Procedure:
Paracentesis

History of Present Illness:
___ 47F w/ HCV cirrhosis c/b ascites, hiv on ART, h/o IVDU, COPD, bioplar, PTSD, presented from OSH ED with worsening abd distension over past week. Pt reports self-discontinuing lasix and spirnolactone ___ 3 weeks ago, because she feels like "they don't do anything" and that she "doesn't want to put more chemicals in her." She does not follow Na-restricted diets. In the past week, she notes that she has been having worsening abd distension and discomfort. She denies ___ edema, or SOB, or orthopnea. She denies f/c/n/v, d/c, dysuria. She had food poisoning a week ago from eating stale cake (n/v 20 min after food ingestion), which resolved the same day. She denies other recent illness or sick contacts. She notes
that she has been noticing gum bleeding while brushing her teeth in recent weeks. she denies easy bruising, melena, BRBPR, hemetesis, hemoptysis, or hematuria. Because of her abd pain, she went to OSH ED and was transferred to ___ HUP for further care. Per ED report, pt has brief period of confusion - she did not recall the ultrasound or bloodwork at

osh. She denies recent drug use or alcohol use. She denies feeling confused, but reports that she is forgetful at times. In the ED, initial vitals were 98.4 70 106/63 16 97%RA
Labs notable for ALT/AST/AP ___ ___: ___, Tbili1.6, WBC 5K, platelet 77, INR 1.6

Past Medical History:
1. HCV Cirrhosis
2. No history of abnormal Pap smears.
3. She had calcification in her breast, which was removed
previously and per patient not, it was benign.
4. For HIV disease, she is being followed by Dr. ___ Dr. ___.
5. COPD
6. Past history of smoking.
7. She also had a skin lesion, which was biopsied and showed skin cancer per patient report and is scheduled for a complete removal of the skin lesion in ___ of this year.
8. She also had another lesion in her forehead with purple discoloration. It was biopsied to exclude the possibility of ___'s sarcoma, the results is pending.
9. A 15 mm hypoechoic lesion on her ultrasound on ___ and is being monitored by an MRI.
10. History of dysplasia of anus in ___.
11. Bipolar affective disorder, currently manic, mild, and PTSD.
12. History of cocaine and heroin use.

Social History:

___
Family History:
She a total of five siblings, but she is not talking to most of them. She only has one brother that she is in touch with and lives in ___. She is not aware of any known GI or liver disease in her family. Her last alcohol consumption was one drink two months ago. No regular alcohol consumption. Last drug use ___ years ago. She quit smoking a couple of years ago.

Physical Exam:
VS: 98.1 107/61 78 18 97RA
General: in NAD
HEENT: CTAB, anicteric sclera, OP clear
Neck: supple, no LAD
CV: RRR,S1S2, no m/r/g
Lungs: CTAb, prolonged expiratory phase, no w/r/r
Abdomen: distended, mild diffuse tenderness, +flank dullness, cannot percuss liver/spleen edge ___ distension
GU: no foley
Ext: wwp, no c/e/e, + clubbing
Neuro: AAO3, converse normally, able to recall 3 times after 5 minutes, CN II-XII intact

Discharge:

PHYSICAL EXAMINATION:
VS: 98 105/70 95
General: in NAD
HEENT: anicteric sclera, OP clear

Neck: supple, no LAD
CV: RRR,S1S2, no m/r/g
Lungs: CTAb, prolonged expiratory phase, no w/r/r
Abdomen: distended but improved, TTP in RUQ,
GU: no foley
Ext: wwp, no c/e/e, + clubbing
Neuro: AAO3, CN II-XII intact

Pertinent Results:
___ 10:25PM GLUCOSE-109* UREA N-25* CREAT-0.3* SODIUM-138 POTASSIUM-3.4 CHLORIDE-105 TOTAL CO2-27 ANION GAP-9
___ 10:25PM estGFR-Using this
___ 10:25PM ALT(SGPT)-100* AST(SGOT)-114* ALK PHOS-114* TOT BILI-1.6*
___ 10:25PM LIPASE-77*
___ 10:25PM ALBUMIN-3.3*
___ 10:25PM WBC-5.0# RBC-4.29 HGB-14.3 HCT-42.6 MCV-99* MCH-33.3* MCHC-33.5 RDW-15.7*
___ 10:25PM NEUTS-70.3* LYMPHS-16.5* MONOS-8.1 EOS-4.2* BASOS-0.8
___ 10:25PM PLT COUNT-71*
___ 10:25PM ___ PTT-30.9 ___
___ 10:25PM ___
.

CXR: No acute cardiopulmonary process.
U/S:
1. Nodular appearance of the liver compatible with cirrhosis.
Signs of portal hypertension including small amount of ascites and splenomegaly.

2. Cholelithiasis.
3. Patent portal veins with normal hepatopetal flow. Diagnostic para attempted in the ED, unsuccessful. On the floor, pt c/o abd distension and discomfort.

Brief Hospital Course:
___ HCV cirrhosis c/b ascites, hiv on ART, h/o IVDU, COPD, bioplar, PTSD, presented from OSH ED with worsening abd distension over past week and confusion.

Ascites - p/w worsening abd distension and discomfort for last week. likely ___ portal HTN given underlying liver disease, though no ascitic fluid available on night of admission. No signs of heart failure noted on exam. This was ___ to med non-compliance and lack of diet restriction. SBP negative
diuretics:
> Furosemide 40 mg PO DAILY
> Spironolactone 50 mg PO DAILY, chosen over the usual 100mg dose d/t K+ of 4.5.
CXR was wnl, UA negative, Urine culture blood culture negative.

Pt was losing excess fluid appropriately with stable lytes on the above regimen. Pt was scheduled with current PCP for ___ check upon discharge. Pt was scheduled for new PCP with Dr. ___ at ___ and follow up in Liver clinic to schedule outpatient screening EGD and ___.

Medications on Admission:
The Preadmission Medication list is accurate and complete.
1. Furosemide 20 mg PO DAILY
2. Spironolactone 50 mg PO DAILY
3. Albuterol Inhaler 2 PUFF IH Q4H:PRN wheezing, SOB
4. Raltegravir 400 mg PO BID
5. Emtricitabine-Tenofovir (Truvada) 1 TAB PO DAILY
6. Nicotine Patch 14 mg TD DAILY
7. Ipratropium Bromide Neb 1 NEB IH Q6H SOB

Discharge Medications:
1. Albuterol Inhaler 2 PUFF IH Q4H:PRN wheezing, SOB
2. Emtricitabine-Tenofovir (Truvada) 1 TAB PO DAILY
3. Furosemide 40 mg PO DAILY
RX *furosemide 40 mg 1 tablet(s) by mouth Daily Disp #*30 Tablet
Refills:*3
4. Ipratropium Bromide Neb 1 NEB IH Q6H SOB
5. Nicotine Patch 14 mg TD DAILY
6. Raltegravir 400 mg PO BID
7. Spironolactone 50 mg PO DAILY
8. Acetaminophen 500 mg PO Q6H:PRN pain

Discharge Disposition:
Home

Discharge Diagnosis:
Ascites from Portal HTN

Discharge Condition:
Mental Status: Clear and coherent.
Level of Consciousness: Alert and interactive.
Activity Status: Ambulatory - Independent.

Discharge Instructions:
Dear Ms. ___,
It was a pleasure taking care of you! You came to us with stomach pain and worsening distension. While you were here we did a paracentesis to remove 1.5L of fluid from your belly. We also placed you on you 40 mg of Lasix and 50 mg of Aldactone to help you urinate the excess fluid still in your belly. As we discussed, everyone has a different dose of lasix required to make them urinate and it's likely that you weren't taking a high enough dose. Please take these medications daily to keep excess fluid off and eat a low salt diet. You will follow up with Dr. ___ in liver clinic and from there have your colonoscopy and EGD scheduled. Of course, we are always here if you need us.
We wish you all the best!

Your ___ Team.

Followup Instructions:
___

LLM Input Example 2

Name: ___ Unit No: ___

Admission Date: ___ Discharge Date: ___

Date of Birth: ___ Sex: F

Service: SURGERY

Allergies:
allopurinol / Statins-Hmg-Coa Reductase Inhibitors

Attending: ___.

Chief Complaint:
Left lower extremity surgical site infection

Major Surgical or Invasive Procedure:
___ Left lower extremity incision and drainage, debridement of left foot ulcer
___ Left lower extremity washout, wound vac placement
___ Left lower extremity wound vac change, debridement
left lower extremity ulcers

History of Present Illness:
Ms. ___ Zaragosa is a ___ 60 year old female recently admitted for management of a chronic, non-healing left foot ulcer who underwent a left femoral to above knee popliteal bypass with NRGSV and left foot ulcer debridement with wound vacplacement. She was seen in clinic 3 days ago with left leg incision healing slowly and evidence of skin separation and wound infection in the left thigh. There was weeping fluid but not purulent and staples intact. There also was significant surrounding erythema and so she was sent to rehab with augmentinand was to follow-up in clinic in 2 weeks. She presents to the ED today with worsening pain and L groin to medial though wound dehiscence and purulent drainage. She is otherwise feeling well without fevers or chills, nausea or vomiting. She was admitted to the vascular surgery service for management of suspected left lower extremity surgical site infection.

Past Medical History:
PMH:
-HTN, labile
-HLD
-HYPOTHYROIDISM
-RETINAL ARTERY OCCLUSION
-Migraine

-CAD/MI (MIs in ___ and ___: This demonstrated a mid

RCA lesion which was stented with a drug-eluting stent. LAD had a proximal 30% stenosis, left circumflex had a ostial 50% stenosis. The distal RCA also had a 50% stenosis)
-CHF (EF 60-65% in ___
-OBESITY
-insulin-dependent DMII
-Gout
-Renal artery stenosis
-CKDIII
-Anemia
-afib
-Depression

PSH:
-Debridement of L foot infected ulcer
-LLE diagnostic angiogram
-L fem-AK pop bypass

Social History:

___
Family History:
Father died of colon cancer in ___.

Physical Exam:
General: NAD
CV: RRR
Pulm: No respiratory distress
Extremities: left groin wound with dressings in place. Bilateral chronic nonhealing ulcers of the lower extremities

Pertinent Results:
ADMISSION LABS:

___ 04:00PM BLOOD Neuts-86* Bands-1 Lymphs-3* Monos-9 Eos-1 Baso-0 ___ Myelos-0 AbsNeut-7.57* AbsLymp-0.26* AbsMono-0.78 AbsEos-0.09 AbsBaso-0.00*
___ 04:00PM BLOOD ___ PTT-53.7* ___
___ 04:00PM BLOOD Glucose-118* UreaN-57* Creat-1.8* Na-139 K-4.8 Cl-97 HCO3-28 AnGap-14

DISCHARGE LABS:

___ 05:46AM BLOOD WBC-11.9* RBC-2.95* Hgb-8.7* Hct-27.5* MCV-93 MCH-29.5 MCHC-31.6* RDW-18.4* RDWSD-59.9* Plt ___
___ 05:46AM BLOOD Plt ___
___ 05:46AM BLOOD ___ PTT-28.0 ___
___ 05:46AM BLOOD Glucose-79 UreaN-68* Creat-2.2* Na-136 K-3.7 Cl-96 HCO3-27 AnGap-13
___ 05:46AM BLOOD Calcium-7.6* Phos-5.3* Mg-2.1

Brief Hospital Course:
Ms. ___ presented on ___ to the emergency room with a concern for a surgical site infection and was given vanc/cipro/flagyl immediately. Her INR was also noted to be 5, so she received 10 of vitamin K in the emergency room. Her repeat INR was 2.3 preop. She was then taken to the operating room in the morning of ___ for a debridement and washout of the LLE. Please see OP note for more details regarding the procedure. Postoperatively, the LLE continued to exsanguinate. Cauterization and compression was done in the PACU and she was transferred to the wards. On ___ evening, she was noted to be hypotensive to SBP ___ and her Hct had drifted from 27 to 21. She was transferred to the SICU for monitoring. She received 2 units of pRBC and 1 unit of FFP along with 10 of vitamin K. Her INR was noted to be 1.7 with Hct stable at 28. Since her last echo was only done in ___, a repeat echo was done that revealed her EF to be 40%, and so she was carefully volume resuscitated in preparation for another debridement, washout and vac placement on ___. Please see op report for more details. Following her ___ postop course, her summary will be written by systems.

#NEURO: Patient was kept intubated and sedated to help facilitate multiple evaluation of her wound, however she was extubated on HD4 due to hypotension. Her pain was controlled with oxycodone and dilaudid.

#CV: Patient was noted to become transiently hypotensive to SBP ___ while she was sedated and so required levo on HD4. Her pressures improved once she was extubated. The patient remained stable from a cardiovascular standpoint; vital signs were routinely monitored.

#PULMONARY: The patient remained stable from a pulmonary standpoint; vital signs were routinely monitored. She was extubated on HD4 without issues.

#GI/GU/FEN: The patient had a foley placed intra-operatively for volume monitoring as well as to keep her incision clean. She was restarted on her home torsemide on ___. The patient was given oral diet once extubated, which she tolerated well. She was noted to have loose bowel movements and incontinent. Her C.diff was negative and so was given a flexiseal on HD3 to keep her wound clean.

#ID: The patient's fever curves were closely watched for signs of infection. She was kept on vanc/cipro/flagyl as her initial wound cultures from her initial washout was noted to be 4+GNR, 2+ GPC in pairs and chains and 1+ GPRs. On HD4, her cultures showed enterococcus and acinetobacter that were resistant to cipro and so was transitioned to ___ on HD4. ID was consulted on HD5. Given that there were no cultures showing MRSA and her vanc trough continued to be high, they recommended holding off on vanc. Her VAC was changed q3d and on her second VAC change, tissue swabs and cultures were sent that showed GPC in chains and pairs and GNRs. Updated culture data suggested VRE and daptomycin was started per ID recs. At the time of her discharge, antibiotics were discontinued according to the patient and her daughter's wishes (see below).

#HEME: Patient received several units of blood over her hospital course for low hematocrits related to bleeding from her left thigh wound. Her last transfusion was ___ and her hematocrits were stable the following two days.

#WOUNDS: The patient's left thigh wound vac was changed every ___ days. She was also found to have bilateral lower extremity pressure ulcers, more extensive on the left than the right leg. The ulcers on the left leg were found to have purulent discharge, so she was taken to the

operating room again on HD9 (___) for debridement of her left lower extremity pressure ulcers. Santyl was used on these ulcers for the first 3 days post operatively. At the time of discharge to hospice, the wound vac was removed and the thigh wound was redressed with wet to dry gauze and overlying curlex.

On ___ a family meeting was held with the patient's daughter and healthcare proxy with a discussion about the lack of progression in her wounds. The following day a second meeting was held with the patient's daughter as well as representatives from palliative care, social work, case management, and vascular surgery. At that time the patient and her daughter elected to transfer the patient to a ___ facility and enact a DNR/DNI order. At the time of her discharge, the patient's vitals were stable.

Medications on Admission:
The Preadmission Medication list is accurate and complete.
1. Acetaminophen 650 mg PO Q8H
2. Albuterol Inhaler 2 PUFF IH Q4H:PRN sob
3. Aspirin 81 mg PO DAILY
4. Bisacodyl ___AILY:PRN constipation
5. BuPROPion (Sustained Release) 150 mg PO QAM
6. Digoxin 0.125 mg PO 3X/WEEK (___)
7. Docusate Sodium 100 mg PO BID constipation
8. Ezetimibe 10 mg PO DAILY
9. Febuxostat 40 mg PO DAILY
10. Ferrous Sulfate 325 mg PO BID
11. Fluticasone Propionate 110mcg 2 PUFF IH BID
12. FoLIC Acid 1 mg PO DAILY
13. HydrALAZINE 20 mg PO Q8H
14. Isosorbide Dinitrate 20 mg PO TID
15. Levothyroxine Sodium 112 mcg PO DAILY
16. Methylprednisolone 4 mg PO DAILY
17. Metoprolol Succinate XL 200 mg PO DAILY
18. Miconazole Powder 2% 1 Appl TP BID
19. nystatin 100,000 unit/gram topical ___ daily
20. Omeprazole 20 mg PO DAILY
21. OxyCODONE (Immediate Release) 10 mg PO Q4H:PRN Pain - Severe

22. Polyethylene Glycol 17 g PO DAILY constipation
23. Prasugrel 10 mg PO DAILY
24. Senna 8.6 mg PO BID:PRN constipation
25. Vitamin D ___ UNIT PO DAILY
26. Metolazone 2.5 mg PO PRN as directed by cardiologist
27. Simethicone 40-80 mg PO QID:PRN bloating
28. Spironolactone 12.5 mg PO DAILY
29. Torsemide 60 mg PO BID
30. Levofloxacin 500 mg PO Q48H foot infection

Discharge Medications:
1. Gabapentin 100 mg PO BID
2. Insulin SC
   Sliding Scale

Fingerstick QACHS, HS
Insulin SC Sliding Scale using REG Insulin
3. OxyCODONE SR (OxyconTIN) 20 mg PO Q12H
RX *oxycodone 20 mg 1 tablet(s) by mouth every twelve (12) hours
Disp #*4 Tablet Refills:*0
4. Acetaminophen 650 mg PO Q8H
5. Albuterol Inhaler 2 PUFF IH Q4H:PRN sob
6. Aspirin 81 mg PO DAILY
7. Bisacodyl ___AILY:PRN constipation
8. BuPROPion (Sustained Release) 150 mg PO QAM
9. Docusate Sodium 100 mg PO BID constipation
10. Ezetimibe 10 mg PO DAILY
11. Febuxostat 40 mg PO DAILY
12. Ferrous Sulfate 325 mg PO BID
13. Fluticasone Propionate 110mcg 2 PUFF IH BID
14. FoLIC Acid 1 mg PO DAILY
15. HydrALAZINE 20 mg PO Q8H
16. Isosorbide Dinitrate 20 mg PO TID
17. Levofloxacin 500 mg PO Q48H foot infection
18. Levothyroxine Sodium 112 mcg PO DAILY
19. Methylprednisolone 4 mg PO DAILY
20. Metoprolol Succinate XL 200 mg PO DAILY
21. Miconazole Powder 2% 1 Appl TP BID
22. nystatin 100,000 unit/gram topical ___ daily
23. Omeprazole 20 mg PO DAILY
24. OxyCODONE (Immediate Release) 10 mg PO Q4H:PRN Pain - Severe
RX *oxycodone 5 mg ___ tablet(s) by mouth every four (4) hours
Disp #*30 Tablet Refills:*0
25. Polyethylene Glycol 17 g PO DAILY constipation
26. Prasugrel 10 mg PO DAILY
27. Senna 8.6 mg PO BID:PRN constipation
28. Simethicone 40-80 mg PO QID:PRN bloating
29. Spironolactone 12.5 mg PO DAILY
30. Torsemide 60 mg PO BID
31. Vitamin D ___ UNIT PO DAILY

Discharge Disposition:
Extended Care

Facility:
___

Discharge Diagnosis:
Surgical site infection of left thigh
Infection of left lower extremity pressure ulcers

Discharge Condition:
Mental Status: Confused - sometimes.

Level of Consciousness: somnolent but arousable.
Activity Status: Out of Bed with lift assistance to chair or wheelchair.

Discharge Instructions:
Dear Ms. ___,

You were admitted to ___ on ___ with a surgical site infection of your left thigh. You were started on antibiotics and taken to the operating room for left thigh debridement and subsequently for placement of a wound vac. You were also found to have left lower extremity pressure ulcers which appeared to be infected, so you were taken back to the operating room for debridement to ensure removal of any dead or infected tissue.

At this time, you have elected to be transferred to a hospice facility. You ongoing care will be under the direction of the hospice team.

Followup Instructions:
___

SUPPLEMENTARY REFERENCES

1. Zhang K, Zhou R, Adhikarla E, et al. A generalist vision-language foundation model for diverse biomedical tasks. Nat Med. (2024). doi: 10.1038/s41591-024-03185-2
2. Xie Q, Chen Q, Chen A, et al. Me LLaMA: Foundation large language models for medical applications. arXiv Preprint. (2024). doi: 10.48550/arXiv.2402.12749
3. Chen Z, Cano AH, Romanou A, et al. MEDITRON-70B: Scaling medical pretraining for large language models. arXiv Preprint. (2023). doi: 10.48550/arXiv.2311.16079
4. Jeong DP, Garg S, Lipton ZC, Oberst M. Medical adaptation of large language and vision-language models: Are we making progress? Proc Emp Nat Lang Proc (2024). doi: 10.48550/arXiv.2411.04118
5. Dorfner FJ, Dada A, Busch F, Makowski MR, Han T, Truhn D, Kleesiek J, Sushil M, Lammert J, Adams LC, Bressem KK. Biomedical large language models seem not to be superior to generalist models on unseen medical data. arXiv Preprint (2024). doi: 10.48550/arXiv.2408.13833
6. Hager P, Jungmann F, Holland R, Bhagat K, Hubrecht I, Knauer M, Vielhauer J, Makowski M, Braren R, Kaissis G, Rueckert D. Evaluation and mitigation of the limitations of large language models in clinical decision making. Nat Med 30: 2613-22. (2024). doi: 10.1038/s41591-024-03097-1
7. Maharjan J, Garikipati A, Singh NP, Cyrus L, Sharma M, Ciobanu M, Barnes G, Thapa R, Mao Q, Das R. OpenMedLM: Prompt engineering can out-perform fine-tuning in medical question-answering with open-source large language models. Sci Rep 14: 14156. (2024). doi: 10.1038/s41598-024-64827-6
8. Lee C, Roy R, Xu M, Raiman J, Shoeybi M, Catanzaro B, Ping W. NV-Embed: Improved techniques for training LLMs as generalist embedding models. Proc ICLR. (2025). doi: 10.48550/arXiv.2405.17428
9. Moreira GSP, Osmulski R, Xu M, Ak R, Schifferer B, Oldridge E. NV-Retriever: Improving text embedding models with effective hard-negative mining. arXiv Preprint. (2024). doi: 10.48550/arXiv.2407.15831
10. Radford A, Wu J, Child R, Luan D, Amodei D, Sutskever I. Language models are unsupervised multitask learners. Open AI Report. (2019). URL: https://github.com/openai/gpt-2
11. Gabarin S. Locutusque/gpt2-large-medical (Revision dd3fb2e). Hugging Face. (2023). doi: 10.57967/hf/1367
12. Jin Q, Dhingra B, Liu Z, Cohen WW, Lu X. PubMedQA: A dataset for biomedical research question answering. Proc EMNLP: 2567-77. (2019). doi: 10.18653/v1/D19-1259
13. Open AI, Achiam J, Adler S, Agarwal S, et al. GPT-4 technical report. arXiv Preprint. (2024). doi: 10.48550/arXiv.2303.08774
14. Jin D, Pan E, Oufattole N, Weng WH, Fang H, Szolovits P. What disease does this patient have? A large-scale open domain question answering dataset from medical exams. Appl Sci 11(14): 6421. (2021). doi: 10.3390/app11146421
15. Johnson AEW, Bulgarelli L, Shen L, et al. MIMIC-IV, a freely accessible electronic health record dataset. Sci Data 10(1). (2023). doi: 10.1038/s41597-022-01899-x
16. Dell'Acqua F. Falling asleep at the wheel: Human/AI collaboration in a field experiment on HR recruiters. Working paper. (2022). URL
17. Dell'Acqua F, McFowland E, Mollick ER, et al. Navigating the jagged technological frontier: Field experimental evidence of the effects of AI on knowledge worker productivity and quality. Harvard Business School Technology & Operations Mgt. Unit Working Paper 24-013. (2023). doi: 10.2139/ssrn.4573321
18. Bastani H, Bastani O, Sungu A, Ge H, Kabakcı Ö, Mariman R. Generative AI without guardrails can harm learning: Evidence from high school mathematics. Proc Nat Acad Sci 122(26); e2422633122. (2025). doi: 10.1073/pnas.2422633122
