# Supplementary material for: Evaluating acute image ordering for real-world patient cases via language model alignment with radiological guidelines
Source: Commun Med (Lond). 2025 Aug 4;5:332. doi: 10.1038/s43856-025-01061-9 (PMC12322208; doi:10.1038/s43856-025-01061-9)
Supplement: Supplementary file 3 — Description of Additional Supplementary Files [file 43856_2025_1061_MOESM3_ESM.pdf]

Description of Additional Supplementary Files

File name- Supplementary Data 1

File description – Source data for all figures.

File name- Supplementary Data 2

File description - Failure Modes of Large Language Models in ACR AC Topic Classification Prediction. We characterized 100 randomly selected instances of incorrect ACR AC Topic predictions made by either Claude Sonnet-3.5 or Llama 3. Qualitatively, we found that there were 5 common failure modes to explain the majority of incorrect predictions made by LLMs: (1) Incorrect prioritization of ACR AC Topics, where life-threatening conditions are overlooked in favor of secondary medical concerns; (2) Fabrication of additional clinical signs or symptoms that were not described in the input patient scenario; (3) Assigning pediatric Topic categories to adult patients or vice versa; (4) Fabrication of semantically related but nonexistent ACR AC Topics; or (5) Incorrect model predictions associated with rare diseases. We report the observed frequency and provide representative examples of each of these failure modes.
