# Supplementary material for: Evaluating acute image ordering for real-world patient cases via language model alignment with radiological guidelines
Source: Commun Med (Lond). 2025 Aug 4;5:332. doi: 10.1038/s43856-025-01061-9 (PMC12322208; doi:10.1038/s43856-025-01061-9)
Supplement: Supplementary file 6 — Reporting Summary [file 43856_2025_1061_MOESM6_ESM.pdf]

nature portfolio

Corresponding author(s): Osbert Bastani (obastani@seas.upenn.edu)

Last updated by author(s): May 12, 2025

Reporting Summary

Nature Portfolio wishes to improve the reproducibility of the work that we publish. This form provides structure for consistency and transparency in reporting. For further information on Nature Portfolio policies, see our Editorial Policies and the Editorial Policy Checklist.

Statistics

For all statistical analyses, confirm that the following items are present in the figure legend, table legend, main text, or Methods section.

n/a	Confirmed	
☐	☒	The exact sample size (n) for each experimental group/condition, given as a discrete number and unit of measurement
☐	☒	A statement on whether measurements were taken from distinct samples or whether the same sample was measured repeatedly
☐	☒	The statistical test(s) used AND whether they are one- or two-sided<br>*Only common tests should be described solely by name; describe more complex techniques in the Methods section.*
☐	☒	A description of all covariates tested
☐	☒	A description of any assumptions or corrections, such as tests of normality and adjustment for multiple comparisons
☐	☒	A full description of the statistical parameters including central tendency (e.g. means) or other basic estimates (e.g. regression coefficient) AND variation (e.g. standard deviation) or associated estimates of uncertainty (e.g. confidence intervals)
☐	☒	For null hypothesis testing, the test statistic (e.g. F, t, r) with confidence intervals, effect sizes, degrees of freedom and P value noted<br>*Give P values as exact values whenever suitable.*
☒	☐	For Bayesian analysis, information on the choice of priors and Markov chain Monte Carlo settings
☒	☐	For hierarchical and complex designs, identification of the appropriate level for tests and full reporting of outcomes
☒	☐	Estimates of effect sizes (e.g. Cohen's d, Pearson's r), indicating how they were calculated

Our web collection on statistics for biologists contains articles on many of the points above.

Software and code

Policy information about availability of computer code

Data collection	Custom code for prospective user study data collection is made publicly available at https://github.com/michael-s-yao/radGPT-UI, and will be available with the manuscript upon publication.
Data analysis	Custom code used for large language model evaluation and data analysis is made publicly available at https://github.com/michael-s-yao/radGPT. All statistical analyses were performed using Python software, version 3.10.13 (Python Software Foundation), the SciPy package, version 1.14.0 (Enthought), and the PyFixest package, version 0.24.2.

For manuscripts utilizing custom algorithms or software that are central to the research but not yet described in published literature, software must be made available to editors and reviewers. We strongly encourage code deposition in a community repository (e.g. GitHub). See the Nature Portfolio guidelines for submitting code & software for further information.

Data

Policy information about availability of data

All manuscripts must include a data availability statement. This statement should provide the following information, where applicable:

- Accession codes, unique identifiers, or web links for publicly available datasets
- A description of any restrictions on data availability
- For clinical datasets or third party data, please ensure that the statement adheres to our policy

All data are available within the article, supplementary information or the source data file provided with this paper. Source data are provided with this paper.

Human research participants

Policy information about studies involving human research participants and Sex and Gender in Research.

Reporting on sex and gender	The gender of both prospective study participants and simulated patient cases for both retrospective and prospective studies are described in Supplementary Tables 3 and 4. Our prospective study evaluates clinician-AI interaction in simulated patient care workflows, which is unlikely to be strongly influenced by study participant sex and/or gender. For this reason, no sex- or gender-based analysis on the prospective study data were performed.
Population characteristics	Study research participants are described in Supplementary Table 4.
Recruitment	Participants for this prospective study were recruited from the Perelman School of Medicine and the Hospital of the University of Pennsylvania where this study was conducted. We provided a monetary incentive of $50 USD to each opt-in, volunteer study participant, and the top 50% most accurate medical students and physicians (scored separately) within each treatment arm were compensated with an additional $10 USD. A total of 23 medical students and 7 resident physicians participated in our experiment; all participating medical students were required to have passed and completed the emergency medicine clinical rotation at the University of Pennsylvania to participate in this study.
Ethics oversight	This study was exempted by the University of Pennsylvania Institutional Review Board (Protocol #856530).

Note that full information on the approval of the study protocol must also be provided in the manuscript.

Field-specific reporting

Please select the one below that is the best fit for your research. If you are not sure, read the appropriate sections before making your selection.

☒ Life sciences ☐ Behavioural & social sciences ☐ Ecological, evolutionary & environmental sciences

For a reference copy of the document with all sections, see nature.com/documents/nr-reporting-summary-flat.pdf

Life sciences study design

All studies must disclose on these points even when the disclosure is negative.

Sample size	For the prospective study involving U.S. medical students and resident physicians, we recruited as many study participants as possible given the constraint imposed by the available budget in financially compensating the volunteer participants. Furthermore, in order to observe a 10% increase in clinician accuracy with LLM-generated assistance in our prospective study, we determined that a sample size of 30 participants (as achieved in our study) is sufficient assuming a Type 1 error rate of 0.05 and power of 0.80. For all other studies not involving human study participants, we used all available data from the RadCases dataset to evaluate LLM performance, which equated to 1,599 unique patient scenarios.
Data exclusions	While constructing the RadCases dataset used to evaluate LLMs, we excluded cases where there exist no ACR AC guidelines relevant to the patient scenario. Such cases might include primary dermatologic conditions and cases where insufficient ACR evidence exist. For our prospective study involving study research participants, no study participants were excluded from analysis.
Replication	All experiments involving large language model inference were conducted a total of 5 different times independently. All relevant statistics reported include the mean and 95% confidence interval over the n = 5 independent experimental runs.
Randomization	For large language model inference experiments, all simulated RadCases patient scenarios were evaluated for each language model, inference strategy, and experiment, and did not require any randomization. The order in which the patient scenarios were evaluated by the language model were randomized based on a random seed. For our prospective randomized control trial, study research participants were randomized to the untimed and timed experimental arms based on a random coin flip.
Blinding	Investigators were blinded to study participant research arm (i.e., untimed or timed) and individual study participant performance and identity during the data analysis relevant for the prospective study described in our manuscript.

Reporting for specific materials, systems and methods

We require information from authors about some types of materials, experimental systems and methods used in many studies. Here, indicate whether each material, system or method listed is relevant to your study. If you are not sure if a list item applies to your research, read the appropriate section before selecting a response.

Materials & experimental systems

n/a	Involved in the study
☒	☐ Antibodies
☒	☐ Eukaryotic cell lines
☒	☐ Palaeontology and archaeology
☒	☐ Animals and other organisms
☒	☐ Clinical data
☒	☐ Dual use research of concern

Methods

n/a	Involved in the study
☒	☐ ChIP-seq
☒	☐ Flow cytometry
☒	☐ MRI-based neuroimaging
